# Influence of Tie-Molecules and Microstructure on the Fluid Solubility in Semicrystalline Polymers

**DOI:** 10.1021/acs.jpcb.2c04600

**Published:** 2022-11-01

**Authors:** Michele Valsecchi, Jona Ramadani, Daryl Williams, Amparo Galindo, George Jackson

**Affiliations:** † Department of Materials, Imperial College London, South Kensington Campus, LondonSW7 2AZ, U.K.; ‡ Department of Chemical Engineering, Imperial College London, South Kensington Campus, LondonSW7 2AZ, U.K.

## Abstract

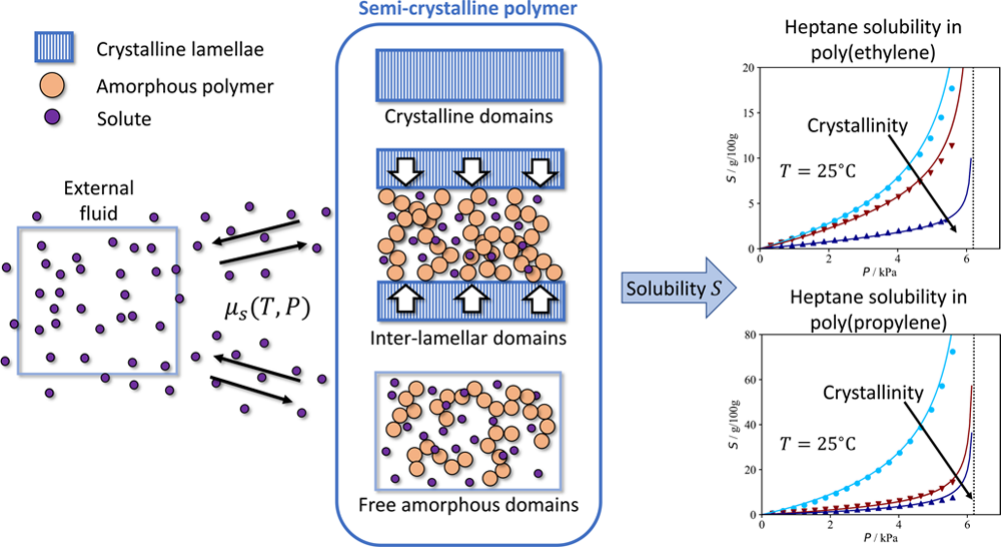

Predicting the absorption of gases and liquids in semicrystalline polymers is of critical importance for numerous applications; the mechanical and transport properties of these materials are highly dependent on the amount of solutes dissolved in their bulk. For most semicrystalline polymers which are in contact with an external fluid, the observed uptake of the solute is found to be lower than that predicted by treating the amorphous domains of the polymer as subcooled polymer melts at the same thermodynamic state. This observation has recently led to the hypothesis that the amorphous domains effectively behave as polymer liquids subject to an additional “constraint pressure” which reduces the equilibrium solubility in the domains. We present a new statistical mechanical model of semicrystalline polymers. The constraint pressure emerges naturally from our treatment, as a property of the interlamellar amorphous domains caused by the stretching and localization in space of the tie-molecules (polymer chains linking different lamellae). By assuming that the interlamellar domains exchange monomers reversibly with the lamellae, the model allows one to simultaneously predict the increase of constraint pressure at low temperatures and the variation of the lamellar thickness as a function of temperature—a phenomenon known as premelting. The sorption isotherms of a range of fluids in different polyethylene and polypropylene samples are determined experimentally and the data is compared with calculations of the new model using the SAFT-VR Mie EoS. In order to accurately predict the absorption close to the vapor pressure of the penetrant, we find that it is essential to include the “free”, unconstrained amorphous domains in the description, resulting in a multiscale model with two adjustable parameters (the fractions of tie-molecules and free amorphous domains) that characterize the morphology of a given semicrystalline polymer sample. The trends observed for the adjusted parameters qualitatively match other estimates reported in the literature.

## 1 Introduction

Modeling and understanding the thermodynamic properties of semicrystalline polymers is one of the most important challenges of polymer physics. In contrast to fully crystalline solids, these materials exhibit very interesting properties such as a temperature-dependent degree of crystallinity, a high-yield strain, and a high-sorption capacity. The origin of these anomalies can be traced back to their peculiar molecular nature and microstructure. The main focus of our current work is to characterize the effect that the microscopic properties of semicrystalline polymers have on the sorption of gases and liquids.

### 1.1 Microstructure of Semicrystalline Polymers

It is useful to start the discussion with a summary of the main features of the semicrystalline “state” of polymeric materials. As their name implies it is convenient to visualize these materials by dividing the total polymer mass into separate crystalline and noncrystalline *amorphous* domains. In practice, this distinction is not always easy to make since neither type of domain can really be regarded as a separate macroscopic thermodynamic phase. Nonetheless, various measures of crystallinity can be defined by using the implicit assumption that some extensive properties of semicrystalline polymers are simply the sum of the respective properties of the two types of domains.

For example, by considering density measurements under the assumption that a unique specific volume *v*_c_ can be assigned to the crystalline domains and a unique specific volume *v*_a_ to the amorphous domains, it is straightforward to show that the mass fraction of crystallinity ω_c_ of a polymer sample (hereon in referred to as crystallinity) is given by^1^
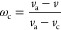
1where *v* is the overall specific volume of the sample. Another measure of crystallinity can be obtained by measuring the enthalpy of fusion of a given sample using differential scanning calorimetry (DSC). Clearly all of these procedures require knowledge of the respective quantities for the “pure” domains, which can be obtained via correlation of experimental data or using a theoretical description. The different assumptions and techniques employed for the calculation of ω_c_ often yield different measures for the crystallinity, thus making this quantity somewhat ill-defined.

Despite this ambiguity, it is possible to recognize the *lamellae* as the smallest crystalline constituents of semicrystalline polymers. These entities are formed by polymer chains folding and stacking, thereby organizing into *quasi*-2D crystalline structures characterized by their average thickness *l*_c_, the lamellar thickness. Depending on the crystallization conditions, the lamellae can organize into bigger mesostructures such as *spherulites* or *shish-kebab* structures.^2,3^ The lamellae do not fill these structures completely: although the precise mechanisms and kinetics of crystallization are still a subject of much debate,^3,4^ it is observed that the lamellae usually stack on top of each other sandwiching layers of amorphous material in between, which are referred to as *interlamellar* amorphous domains. These alternating structures (the so-called “lamellar stacks”) lack perfect order, but it is often possible to assign a corresponding average interlamellar thickness *l*_a_ to these amorphous layers.

Lamellar stacks are not, however, the only constituent of semicrystalline polymers. Some portions of a given mesostructure may be devoid of any lamella, and if the crystallinity is low enough, it is possible to find “free” amorphous regions of this type outside of the mesostructure as if the latter were embedded in an amorphous polymer matrix. These observations question the notion that all the amorphous mass can be treated in the same way; it has been shown in recent studies^5^ that it is possible to further divide the total amorphous mass into a “constrained”, rigid portion (presumably composed of the interlamellar amorphous mass) and a “free”, unconstrained portion by measuring the transverse relaxation time of polymer chains via low-field ^1^H NMR. As expected, one finds that the mass fraction ψ of “free” amorphous domains relative to the total polymer mass decreases with increasing crystallinity, since at high crystallinity the lamellar stacks fill the whole volume of the semicrystalline polymer.

### 1.2 Sorption in Semicrystalline Polymers

If a semicrystalline sample is in equilibrium with a pure external fluid at temperature *T* and pressure *P*, the total sorption *S* of the fluid in the polymer sample—measured in grams of fluid retained by the sample per gram of pure polymer—is the sum of an *adsorption* term on the surface of the polymer and a bulk *absorption* term:
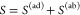
2In practice, however, unless the sample has a very high specific surface or the absorption is insignificant, it can be assumed that the total sorption is almost entirely represented by absorption, i.e., *S* ≈ *S*^(ab)^. In the following analysis, only the absorption is considered. One of the most common starting assumptions employed to model *S* ≈ *S*^(ab)^ at given *T*, *P* conditions is that the crystalline domains are essentially impermeable to the solutes relatively to the amorphous ones; mathematically, this translates as
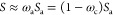
3where *S*_a_ represents the absorption in the amorphous domains only. Experimental evidence supporting the idea that crystalline domains are impermeable to the solute molecules has been available since the studies of Michaels and co-workers^6−8^ on the solubility of simple gases in polyethylene. Heuristically, this behavior can be explained by comparing the high enthalpy of formation of a defect in the crystalline phase with the enthalpy of solution in the amorphous domains; it is energetically unfavorable for a solute to deform a dense, ordered lattice rather than mixing in the less dense and already disordered amorphous domains.

It should be noted, however, that there are some notable exceptions to this general trend. Polymers with very bulky monomers and with high stereoregularity (such as sindiotactic polystyrene, s-PS) can in fact form crystal structures which are able to accommodate solute particles as interstitials without deforming the crystal architecture significantly, thereby reducing the enthalpy of formation of a defect up to the point where *ad*sorption in the interstitial sites becomes favorable.^9−11^ Another exception arises when the particles absorbing are very small, as is the case for molecular hydrogen and helium. In the ensuing discussion, we focus on systems for which the approximation embodied in eq 3 is justified.

In our current work, a series of sorption experiments on semicrystalline polyethylene and polypropylene samples is performed and subsequently analyzed using a newly developed model characterizing the effects of the crystallinity and the internal morphology on the thermodynamic properties of semicrystalline polymers. In the following section, the experimental procedure to obtain the sorption isotherms and crystallinity of the samples is presented. The model is developed in Section 3 after a survey of previous work. Sections 4 and 5 are dedicated to a critical review of the theoretical representation of the experimental data and conclusions.

## 2 Experimental Methods

### 2.1 Materials

All the substances used for the generation of vapor isotherms—namely *n*-hexane, *n*-heptane, and cyclohexane—were ordered from Sigma-Aldrich (Poole, U.K.) and VWR U.K. with a minimum of 99% purity. These reagents were used without further purification. The deionized (DI) water used for all the experiments was ultrapure Milli-Q grade. All the polyethylene samples used in our work were ordered from Sigma-Aldrich (Poole, UK) and all the polypropylene samples were ordered from Sp2 Scientific Polymer Products Inc. (NY, USA). Isotactic polypropylene (iPP) was received as pellets, atactic polypropylene (aPP) as a waxy solid, low-density polyethylene (LDPE) and high-density polyethylene (HDPE) as pellets, and medium-density polyethylene (MDPE) as a fine powder. The commercial nonwoven (isotactic) polypropylene fibers (fPP) used in our research were donated by Procter & Gamble.

### 2.2 Sample Fabrication

Apart from aPP, which was dissolved in toluene at room temperature, all the other polymers were dissolved in decahydronaphthalene at 160 °C to form 0.02 mg cm^−3^ solutions. An alumina foil swatch (diameter 6.5 cm) was precleaned with DI water and 2-propanol, dipped into the polymer solution for 30 s, and then left to air-dry for 30 min. The films obtained were then placed in a vacuum oven for 3 h at 80 °C for aPP and 120 °C for the other samples in order to evaporate the remaining solvents. Each alumina foil preparation was weighed before and after polymer coating to record the amount of polymer film created.

### 2.3 Dynamic Vapor Sorption (DVS): Instrumentation

The sorption profiles of the polymer films were determined using the DVS Endeavor and Resolution (Surface Measurement Systems, London, U.K.). The samples, with mass ranging between 100 and 140 mg, were first directly hung on the DVS chamber’s hang-down hook, and the sample pan was removed. Before the sorption cycles, the samples were dried at 0% relative humidity (RH) and 25 °C for 180 min to establish a dry mass. Counterweights were used for the higher mass samples on the DVS Resolution. Most samples were folded into smaller units to keep them compact. A series of experiments was then carried out using either fixed times for each experimental humidity set point or using a %d*m*/d*t* threshold mode. Humidity or RH here refers to the ratio between the partial pressure of the target solute and its vapor pressure at the corresponding temperature.

In the %d*m*/d*t* mode, the percentage change of mass with time is measured and compared to a threshold value to determine the equilibration time at each given RH step. The %d*m*/d*t* threshold was set to 0.0005% for all experiments to ensure the sample had reached a necessary degree of equilibrium before moving on to the next step. When the sample percentage change in mass was equal to or below this threshold for 10 min, the step stage was ended and moved onto the next programmed RH% step. Methods were run in (0–90% RH) cycles with increments of 10% RH steps. Partial pressures were generated using liquid solvent bubbling reservoirs and controlled via closed-loop speed of sound sensors. In a first series of experiments, the temperature was set to 25 °C and the sorption cycles of each individual solute were measured in all six polymer samples. n-Heptane sorption cycles were also measured at 35, 45, and 55 °C.

Sorbed quantities were calculated using the change in mass between the ends of the current cycle’s sorption and previous cycle’s desorption step. A flow rate of 200 cm^3^ min^−1^ was used for all experiments, with the carrier gas being nitrogen in all cases. Between experiments, samples were dried at 50 °C under vacuum for 3 h to remove any residual solute or other contaminants that may influence sorption performance. The raw data were exported into Microsoft Excel, and the analysis was undertaken using the DVS Macro Standard Analysis Suite v7.0.13 (Surface Measurement Systems, London, U.K.).

### 2.4 Density Measurement: Helium Pycnometry Instrumentation

The density of the dried polymer samples was measured at 25 °C and 1 bar in order to determine their mass fraction of crystallinity ω_c_ using eq 1. The correlated densities of fully amorphous and fully crystalline polymers appearing in eq 1 were taken from the literature,^12,13^ except for the density of fully amorphous PP, which was taken to be equal to the density of atactic PP. At 25 °C for PE, ρ_a_ = 0.852 kg dm^–3^, and ρ_c_ = 1.000 kg dm^–3^; for isotactic PP, ρ_a_ = 0.840 kg dm^–3^, and ρ_c_ = 0.946 kg dm^–3^. The measurements were carried out via pycnometry using an Accupyc II 1340 (Micromeritics, USA) instrument, with helium gas as the probe molecule. The densities and related crystallinity of the dried samples are reported in Table 1.

**Table 1:** Density ρ and Mass Fraction Crystallinity ω_c_ at 25 °C and 1 bar of the Dried Samples Obtained after Solution Casting on the Alumina Foils^a^

	LDPE	MDPE	HDPE	aPP	fPP	iPP
ρ(25 °C)/(kg dm^–3^)	0.916	0.917	0.920	0.840	0.883	0.899
ω_c_(25 °C)	0.472	0.479	0.499	0	0.435	0.586

a The crystallinity was calculated using eq 1.

Since the density (and therefore crystallinity) was measured for the solution-cast samples, its value differed from the one reported by the supplier. In particular, all three polyethylene samples had a similar crystallinity despite having a markedly different sorption capacity—see the Results section.

## 3 Modeling Sorption in Semicrystalline Polymers

If adsorption is neglected the total sorption *S* in a semicrystalline polymer can be taken to be the ratio of the total absorbed solute mass *m*_s_ and the total polymer mass *m*_p,tot_. Since here the crystalline polymer is assumed to be impermeable to the solute, by taking *m*_s,a_^F^ and *m*_s,a_^IL^ as the mass of solute in the free and interlamellar domains, respectively, we can write
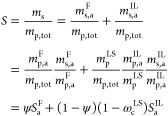
4Here, ψ is the fraction of free amorphous domains in the total polymer mass, and ω_c_^LS^ = *m*_c_/(*m*_c_ + *m*_p,a_^IL^) is the crystallinity of the lamellar stacks (LS)—since all crystalline polymers are considered to be lamellar. The sorption in the free and interlamellar amorphous domains (*S*_a_^F^ = *m*_s,a_^F^/*m*_p,a_^F^ and *S*_a_^IL^ = *m*_s,a_^IL^/*m*_p,a_^IL^, respectively) are calculated as the ratio of the mass of solute and the polymer mass in the respective domain. By comparing eq 4 to eq 3, it is easy to show that
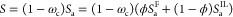
5where ϕ = *m*_p,a_^F^/(*m*_p,a_^F^ + *m*_p,a_^IL^) is the mass fraction of free amorphous mass relative to the total amorphous mass in the sample; the total sorption in the amorphous domains thus satisfies *S*_a_ = *ϕS*_a_^F^ + (1 – ϕ) *S*_a_^IL^. This is the first time more than one type of amorphous domain has been considered in order to model sorption in semicrystalline polymers. In previous work in the area, the amorphous domains were considered to be homogeneous and characterized by a single value of *S*_a_.

### 3.1 Experimental Evidence: Constrained Amorphous Domains

From a thermodynamic perspective, the equilibrium between a pure external fluid and the free or interlamellar amorphous domains with respect to exchanges of solute particles is realized through the equality of the chemical potential μ of the solute in the three systems:
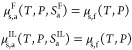
6Here, the subscript s indicates the solute, and the subscripts a and f indicate the amorphous domain and external fluid phase, respectively. It is important to note that the external fluid phase is assumed to be pure, as polymers have vanishingly small vapor pressure. Furthermore, for simplicity here, the composition of the amorphous domains is only specified through the solubility in the amorphous phase *S*_a_ instead of the mole fraction of solute as the molecular weight of the polymer chains in the amorphous domains may vary. This choice is justified by the weak dependence of the vapor–liquid equilibrium (VLE) of polymer mixtures on the polydispersity and the mean molecular weight of the polymer.^1^

In order to solve eq 6 for the solubilities *S*_a_^F^ and *S*_a_^IL^, it is necessary to specify the chemical potential of the solute in the three phases. The simplest approach to this challenge is to assume that, due to their disordered nature, the amorphous domains behave as subcooled polymer melts which can be treated as a liquid; this implies that the value of *S*_a_ = *S*_a_^F^ = *S*_a_^IL^ can be obtained directly from a VLE calculation using an equation of state (EoS) for fluids that can describe the properties of both the external fluid and of the polymer–solute mixture above the melting temperature of the polymer:
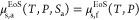
7Here, the superscript EoS indicates that the functional form of the chemical potential is imposed by the choice of a given equation of state. The solution of eq 7 should not depend significantly on the molecular weight of the polymer at medium to high molecular weights due to the considerations mentioned earlier,^1^ and thus the calculation can be performed with an arbitrary molecular weight corresponding to, e.g., 1000 repeating units. In the following analysis, the solubility of the solute in the amorphous polymer calculated through eq 7 is denoted with *S*_a_^EoS^(*T*,*P*). This is the approach originally developed by Michaels and Bixler,^6^ who calculated the chemical potentials of the polymer-solute mixture using the Flory–Huggins–Staverman theory.^14−16^

**Figure 1:**
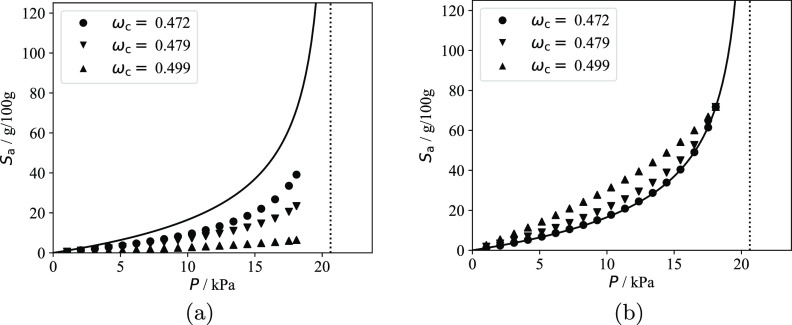
Solubility of *n*-hexane in the amorphous domains of semi-crystalline polyethylene at 25 °C. The continuous curves represent SAFT-γ Mie calculations using the model of Papaioannou et al.^17^ for *n*-hexane and polyethylene (1000 CH_2_ units).^18^ The symbols represent experimental data (uncertainty smaller than the marker size), while the vertical dotted lines represent the vapor pressure *P*_vap_ of *n*-hexane at 25 °C. (a) Comparison of the theoretical prediction *S*_a_^EoS^ and the apparent amorphous solubility *S*_a_^exp^ in the three samples calculated using eq 8. (b) Comparison of the theoretical prediction and the scaled solubility in the three samples; a linear scaling is performed by enforcing that the apparent solubility in the three samples at *P* = 0.9*P*_vap_ matches the theoretical predictions.

It is evident from a number of studies^19−21^ that the apparent solubility in the amorphous domains *S*_a_^exp^ is lower than *S*_a_^EoS^, regardless of the equation of state used. The apparent solubility of *n*-hexane in the amorphous domains of the three polyethylene samples measured at 25 °C is compared in Figure 1a with *S*_a_^EoS^ calculated using the SAFT-γ Mie model for linear alkanes developed by Papaioannou and co-workers;^17,18,22^ more details are provided at the beginning of Section 4. The apparent experimental amorphous solubility *S*_a_^exp^ is obtained from eq 3 via the relationship
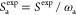
8where *S*^exp^ is the measured solubility.

One can see from Figure 1a that *S*_a_^exp^ < *S*_a_^EoS^ for all of the semicrystalline samples. In particular, the apparent amorphous solubility is found to decrease with increasing crystallinity in the semicrystalline samples. The same behavior is observed for other solutes and in the three polypropylene samples studied. One can justify these findings while assuming *S*_a_ = *S*_a_^EoS^ if either the calculated crystallinity greatly underestimates the true value or if only a fraction of the amorphous domains is available for sorption, resulting in an “effective” crystallinity which is higher than the calculated value.

Fortunately, it is possible to test the validity of these assumptions. If either of these hypotheses are correct, a linear scaling of *S*_a_^exp^ at each pressure so that the apparent amorphous solubilities in different polyethylene samples match at a given pressure should result in three overlapping experimental curves. Physically, the linear scaling is equivalent to assuming that the calculated crystallinity used in eq 8 is underestimated. In Figure 1b, the three experimental data sets are scaled so that the amorphous solubility measured at the highest pressure (i.e., 90% of the vapor pressure of *n*-hexane at 25 °C) matches the theoretical calculations. It is apparent that the scaled experimental data sets do not match and that the curvature decreases with increasing crystallinity.

These findings clearly indicate that the solubilities in the amorphous domains of the three different PE samples are different. In particular, if the measured values of crystallinity are accepted as being correct, it follows that *S*_a_^exp^ < *S*_a_^EoS^ for all samples and that *S*_a_^exp^ decreases with increasing crystallinity.

### 3.2 Constraint Pressure Formalism for Swollen Networks

In an effort to justify the observed reduction of the solubility of simple gases in polyethylene, Memari and co-workers^20,23,24^ proposed that the constraining effect of the crystals make the amorphous domains of semicrystalline polymers—hereafter treated as a homogeneous amorphous mass, temporarily neglecting the difference between free and interlamellar domains—behave as an unconstrained liquid subject to an extra isotropic stress, the “constraint pressure” *P*_c_, which increases the effective thermodynamic pressure in those domains from the external pressure *P* to *P* + *P*_c_. The same idea was employed by Minelli and co-workers^21^ to calculate the solubility of simple gases in PE, PP, and poly(ethylene oxide) (PEO), using the Sanchez–Lacombe equation of state.^25^ The chemical potential of the solute in the amorphous domains is thus written as
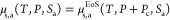
9Both authors found that the typical values of *P*_c_ needed to correctly describe the sorption isotherms ranged between 10 and 100 MPa, a very significant pressure compared to the typical external pressure.

Some authors believe that the constraint pressure is of a mechanical origin, either due to potential residual stresses in the polymer following crystallization or due to the formation of cavities needed to accommodate the solute particles in the amorphous domains.^26^ We take another perspective in our current work, namely that the constraint pressure originates from the elastic forces exerted by the tie-chains and tie-entanglements^27^ on the interlamellar amorphous domains and from the localization in space of the polymer segments. This hypothesis stems from the observation that in cross-linked polymer networks (such as rubbers and gels) the bulk solubility is lower than the one measured for non-cross-linked polymers. It is now well understood^28−30^ that this phenomenon can be traced back to the increase in the free energy of the chain segments between cross-links following the expansion (or “swelling”) of the network caused by the introduction of a penetrant.

Interestingly, theories for swelling in rubbers and semicrystalline polymers have never incorporated a constraint pressure explicitly as they have been developed in terms of expressions for the excess chemical potential *Δμ*_s_^c^ due to the presence of the cross-links compared to the unconstrained state. We show here that both approaches are equivalent. The free energy of a swollen network *A* can be written as
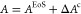
10where *A*^EoS^ is the reference free energy of a liquid polymer-solute mixture and *ΔA*^c^ the free-energy difference due to network constraints (e.g., chemical or physical cross-links). This free-energy difference is mostly entropic in origin and due to the reduced configuration space available to polymer chains upon cross-linking and deformation.^31^ Let *T*, *V*, ***n***_s_, **ν**, and **Γ**_c_ be, respectively, the temperature, the volume, the solute(s) composition vector, the polymer composition vector (i.e., the molecular weight distribution), and the vector of variables specifying the constraints acting on the reference liquid. Since these constraints are generally assumed to be permanent, the pressure is obtained as
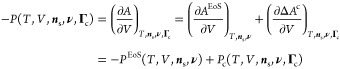
11where *P*^EoS^ is the pressure of the reference unconstrained liquid, and we have defined
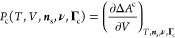
12This quantity is positive since the configurational entropy of the network chains invariably decreases upon increases in volume. By rearranging eq 11, we see that at constant pressure *P* the equilibrium volume *V*(*T*, *P*, ***n***_s_, **ν**, **Γ**_c_) is a solution of
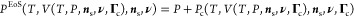
13Since the functional relationship between *P*^EoS^ and *V* is invertible, we then have
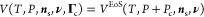
14where *V*^EoS^ is the equilibrium volume of the reference liquid and *P*_c_ is a function of state via eq 12. Simultaneous solution of eqs 12 and 14 yields the equilibrium values of *V* and *P*_c_ at fixed *T*, *P*, ***n***_s_, **ν**, and **Γ**_c_.

In the vast majority of previously developed network models,^28,30,32^ the polymer chains statistics in the reference polymer liquid is assumed to be unperturbed by the presence of the solute—the so-called phantom-chain statistics^31^—resulting in a free-energy difference *ΔA*^c^ which does not depend explicitly on the number of moles of solute ***n***_s_:
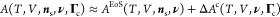
15It must be stressed that this hypothesis is not justified for highly swollen networks (e.g., gels) far from their θ temperature.^33^ Under this approximation, the chemical potential μ_s,*i*_ of a solute *i* is given by
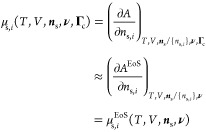
16

At fixed pressure *P*, exploiting now the invertibility of the function μ_s,*i*_^EoS^(*V*) and using eq 14 we finally obtain
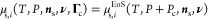
17It is useful to note the similarity to eq 9, aside from the difference in how the composition is specified (*S*_a_ versus ***n***_s_, **ν**). The combination of eqs 14 and 17 elucidates how, for systems in which *ΔA*^c^ is of the form of eq 15, the effect of constraints is *formally* equivalent to the addition of a (state-dependent) pressure *P*_c_ to an otherwise unconstrained liquid polymer-solute mixture.

Since the “liquid-like” amorphous domains have a very low compressibility, the increase in chemical potential of a solute *i* due to the presence of network constraints can be expressed as
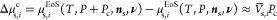
18where  = (*T*, *P* + *P*_c_, ***n***_s_, **ν**) is the partial molar volume of the penetrant in the bulk rubber or amorphous domains—a quantity that is in principle a function of state but is sometimes taken to be constant. All theories in which one assumes *ΔA*^c^ to be composition-independent can thus be unified under the aforementioned formalism. The magnitude of the constraint pressure can be calculated from eq 18 by simply dividing the excess chemical potential obtained with these models by 

Due to the similarity between the chemical cross-links in rubbers and the physical cross-links between tie-chains and lamellae in semicrystalline polymers, theories for swelling in rubbers such as the Flory–Rehner theory^28^ and the Sanchez–Lacombe theory modified for network structures^30^ have indeed been applied to semicrystalline polymers, providing good agreement with the experimental data for sorption of hydrocarbon vapors in semicrystalline polypropylene^34^ and of supercritical CO_2_ in semicrystalline polytetrafluoroethylene (PTFE), perfluoroalkoxy polymer (MFA), and polyvinylidene difluoride(PVDF).^35^ The use of these theories for the description of sorption in semicrystalline polymers nevertheless overlooks important differences between these materials and rubbers. First of all, most of these models (with some exceptions^32,36^) assume an isotropic swelling of the amorphous polymer mass despite the clear anisotropy of the constrained interlamellar amorphous domains. Furthermore, the Gaussian approximation for the end-to-end probability distribution of the chain end-to-end distance (which is valid for small displacements) is employed, despite evidence suggesting that the tie-chains and tie-entanglements in semicrystalline polymers are actually very taut^37^ at ambient conditions. Both these approximations are relaxed in our model (see Section 3.4).

### 3.3 The Local Equilibrium Hypothesis

**Figure 2:**
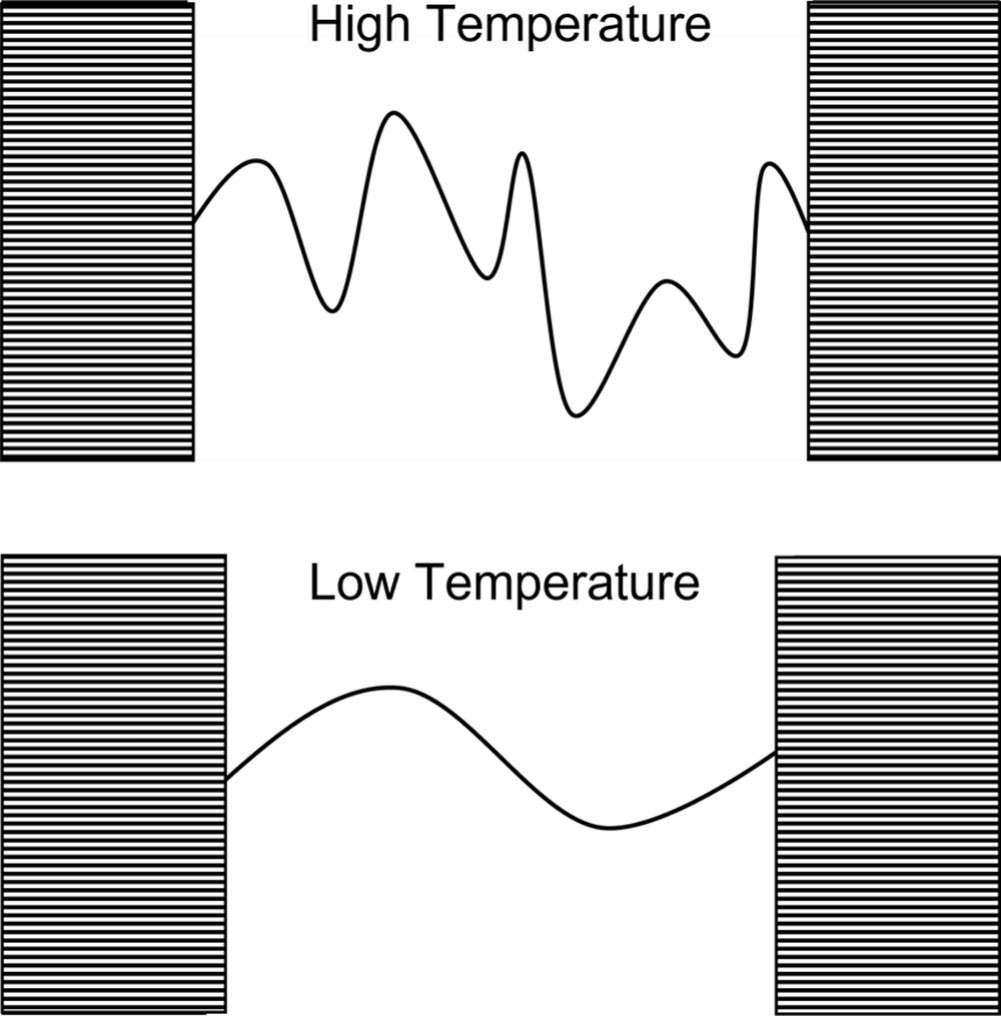
Schematic illustration of the local-equilibrium hypothesis: the tie-chains are shorter and more taut at low temperatures due to the increase in the driving force of crystallization. The lamellae (rectangles) are thicker at lower temperatures due to the inclusion of more tie monomers.

The network models discussed usually require the specification of the average length of the chain segment between cross-links (or, equivalently, the cross-link density) as a free parameter. Michaels and Hausslein (MH)^19^ were among the first authors to notice that the excess elastic activity *a*_s,*i*_^c^ = exp(*Δμ*_s,*i*_^c^ /*RT*) of various penetrants in polyethylene depends markedly on temperature, with a reversible behavior below about 100 °C. In order to explain this phenomenon using the Flory–Rehner theory, the cross-link density must vary with temperature: this is not consistent with the reversibility of the phenomenon, as the detachment of one tie-chain or loop from a given lamella is an irreversible process. Michaels and Hausslein then proposed that (in polyethylene) each tie-molecule is in equilibrium with the crystalline lamellae with respect to exchanges of monomers. This phenomenon makes the tension of the ties temperature dependent, as it is the result of a local equilibrium between the entropic forces that “loosen” the chains and the driving force of crystallization which, by contrast, gives rise to taut chains as a result of the removal of monomers (see Figure 2). Hereafter, their hypothesis is thus referred to as the “local-equilibrium hypothesis”. Using this assumption, they developed a model for the excess elastic chemical potential of a penetrant featuring the mass fraction *f*_T_ of elastically effective chains (i.e., tie-chains and entangled loops) in the amorphous domains as a free parameter.

The resulting MH theory has been applied extensively to model the sorption of various compounds in PE^19,34,38−44^ and PE copolymers.^45^ However, just like the network model mentioned in the previous section, in the MH theory one assumes isotropic swelling of the amorphous domains and employs the Gaussian approximation for the end-to-end probability distribution of the chain segments, despite the main result of the theory predicting that the ties should be very taut at ambient conditions (as is shown later in our current work). Furthermore, since the equilibrium number of tie-monomers is temperature-dependent, the mass fraction of elastically effective chains *f*_T_ should also vary with temperature, whereas it has been taken as constant for each polymer sample in all subsequent work.

It is important to understand which semicrystalline polymers possess this behavior, as clearly the existence of the local equilibrium at the lamellar surface has important implications on the nature of the interlamellar amorphous domains. Some authors have argued that in semicrystalline polymers such as PE and PEO, the high mobility of the chain might allow the lamellae to reorganize,^4,46,47^ thus potentially activating the molecular mechanisms that justify the local equilibrium between the lamellae and the ties. For other polymers, however, the mechanism of partial chain crystallization as the basis of the theory might simply be inactive due to kinetic and/or thermodynamic constraints. The lamellar thickness of sPP, for example, appears to remain roughly constant with temperature.^4^ Some authors have postulated that each polymer can be classified either as “crystal-mobile” or “crystal-fixed” based on the presence of the α_c_-relaxation mode observed in NMR experiments.^3,48^

The local equilibrium hypothesis was used in another context by Fischer^49^ and later studies^50−52^ in order to explain the reversible changes of the lamellar thickness with temperature observed in PE,^53^ a phenomenon known as premelting. In the earliest theoretical attempts,^49,50^ the interlamellar amorphous domains are assumed to be composed of non-entangled loops, and the resulting empirical parameters of the models had unphysical values.^52^ More recently, Albrecht and Strobl^52^ assumed that these domains are, by contrast, only composed of a network of entangled segments, treating the network junctions (i.e., the entanglement points between two loops) as slip-links. This approach allowed the authors to account for a “stretching contribution” of the entangled segments following changes in the interlamellar distance, successfully describing the variation of the interlamellar distance with temperature. Nonetheless, this model still employs the Gaussian approximation inconsistently.

Interestingly, none of these studies appear to acknowledge the earlier work by Michaels and Hausslein, possibly because their theory focused on solubility rather than melting. Despite the slightly different assumptions in the models, however, it is clear that the same physical picture of the interlamellar amorphous domains has been used to characterize two different properties of semicrystalline PE, namely premelting and sorption behavior (i.e., the temperature dependence of constraint pressure). In fact, it should be possible to use the Albrecht and Strobl model to calculate the constraint pressure exerted by the newtork of taut entangled segments on the interlamellar amorphous domains of the PE sample they studied.

We will now develop a general formalism to explore the consequence of the local-equilibrium hypothesis on the thermodynamic properties of pure and swollen polymer networks. Let us assume again that the free energy of a constrained polymer system satisfies eq 15. Furthermore, for simplicity let us assume that the constrained polymer system is made of ν identical chains of *n* monomers each and *n*_s_ solute molecules of a single species. The following arguments developed here can be readily extended to polydisperse chain distributions. In the following, we thus drop the bold notation for the number of chains and solute molecules. Since *n* can change it is hereon included in the variables characterizing *A*. We will further write *A* = *A*(*T*, *V*, *n*_s_, *n*; ν, **Γ**_c_) to emphasize that the constraints **Γ**_c_ and therefore ν cannot vary. Equilibrium with respect to exchanges of chain monomers between the system and a reservoir—in this case, the crystalline lamellae—requires that
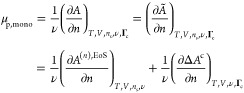
19where we have defined μ_p,mono_—the “monomer chemical potential”—as the change in free energy of the lamellae due to the incorporation of one monomer. The superscript (*n*) has been added to *A*^EoS^ to emphasize that in the reference liquid mixture the polymer molecules have *n* monomers. We have also defined *Ã* = *A*/ν as the free energy per polymer chain.

Both derivatives in the last line eq 19 can be performed once the functional dependence of the free energies on *n* is specified. However, it might be impractical or impossible to differentiate *A*^(*n*),EoS^ with respect to *n* as we are treating the latter as a continuous variable, whereas real polymer chains can only have an integer number of monomers. In order to overcome this issue, we exploit the intuitive concept that the intensive properties of the unconstrained polymer–solute mixture—with the exception of the chemical potential of the polymer—should not depend on the molecular weight of the polymer as long as the latter is large enough and the mass fraction of the solute is kept constant. This procedure will allow us to take the derivatives with respect to *n*, as is shown in Appendix A.2.

For all numerical calculations, the intensive properties of the unconstrained polymer mixture are then calculated using a reference mixture in which polymer chains have an arbitrary constant number of monomers *n*_0_ and where the composition is chosen to ensure that the mass fraction of solute is the same as the unconstrained mixture. This implies that for all intensive properties the superscript will be changed from *n* to *n*_0_, and the composition will be modified as follows:
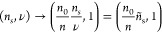
20where *ñ*_s_ = *n*_s_/ν. The dependence on *n* of the thermodynamic properties is now enforced through the composition only. We can now continue with our discussion. eq 19 determines the equilibrium number of chain monomers at fixed *T*, *V*, *n*_s_, and μ_p,mono_. If the pressure *P* is fixed instead of the volume, the equilibrium value of *n* is instead found by solving
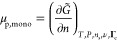
21where *G̃*(*T*, *P*, *n*_s_, *n*; ν, **Γ**_c_) = *Ã* + *PV*(*T*, *P*, *n*_s_, *n*; ν, **Γ**_c_)/ν is the reduced Gibbs free energy. The equilibrium volume is here explicitly a function of *n* and is found from the simultaneous solution of eqs 12 and 14. If the chemical potential of the solute μ_s_ is also fixed by an external fluid, the equilibrium number of solute molecules *n*_s_ is found by solving
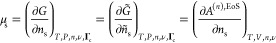
22where we have used eq 16 in the last equality. We note that the conditions expressed by eqs 21 and 22 are equivalent to imposing that at fixed *T*, *P*, μ_s_, and μ_p,mono_, the reduced potential
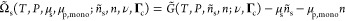
23is stationary with respect to *ñ*_s_, *n*.

At fixed *T*, *P*, μ_s_, and μ_p,mono_ the combination of eqs 13, 17, 21, and 22 thus determines the equilibrium volume *V*, the reduced number of solute molecules *ñ*_s_, and the number of chain monomers *n*. This very general procedure can be applied to any constrained polymer system satisfying the requirements of eq 15. One should point out that applying simultaneously the four driving forces *T*, *P*, μ_s_, and μ_p,mono_ is not at odds with the Gibbs–Duhem equation for the constrained systems as its free energy *A* is in general not extensive in all the four respective variables *S*, *V*, *n*_s_, and *n* due to the presence of the term *ΔA*^c^.

Now that the formalism has been developed, it is sufficient to specify

- the form of the free energy difference *ΔA*^c^(*T*, *V*, *n*; ν, **Γ**_c_),
- the form of the volume *V*(*T*, *P*, *n*_s_, *n*; ν, **Γ**_c_), and
- the dependence of μ_s_ and μ_p,mono_ on the external temperature *T* and pressure *P*

to completely specify the problem. The next section is thus dedicated to developing models for the free energy of the interlamellar amorphous domains, *A*^IL^, and of the free amorphous domains, *A*^F^.

### 3.4 Free Energy of the Amorphous Domains

#### 3.4.1 Free Amorphous Domains

We treat the free amorphous domains as subcooled polymer + solute mixtures (*A*^F^ ≈ *A*^EoS^) due to the apparent absence of constraints in these domains.^5^ Although we can expect inhomogeneities in both the density and stress to be present in these domains, this assumption reflects the defining property of free amorphous domains, i.e., being composed of “loose” polymer chains with slow transverse relaxation.^5^ Consequently *S*_a_^F^ at each temperature and pressure is calculated using eq 7 (i.e., *S*_a_^F^(*T*, *P*) = *S*_a_^EoS^(*T*, *P*)). It should be noted that we assume the local equilibrium between the free amorphous mass and the lamellae to be absent; this is a crude simplification, as the persistence of amorphous mass in these domains must be due to local equilibrium on the lateral lamellar surfaces^4,54^ or the global thermodynamic effect, due for example to chain defects.^1,55,56^

However, this assumption is necessary if we take *A*^F^ = *A*^EoS^ since otherwise, below the melting point of the polymer, all the monomers of the polymer chains would be incorporated in the lamellae due to the absence of constraints. While this approximation should not impact significantly the sorption behavior of free amorphous domains (see eq 17 and Section 4), it may be important to relax it if the variation of the free amorphous mass with temperature and concentration needs to be calculated.

#### 3.4.2 Interlamellar Domains

In this section, an expression for the free energy of the interlamellar amorphous domains in semicrystalline polymers is derived, based on a simple statistical-mechanical model. The constraint pressure emerges naturally from this treatment as a property of the interlamellar domains due only to the presence of tie-chains and tie-entanglements bridging the two opposing lamellae. The local-equilibrium hypothesis is then applied in order to remove an unknown from the problem (namely, the average number of monomers of the ties). The resulting model is characterized by two free parameters: the fraction of stems *p*_T_ connected to tie-chains or entangled loops, and the interlamellar distance *l*_a_^*^ of the pure semicrystalline polymer at an arbitrary temperature T* and pressure P*. This allows one to calculate the dependence of both *P*_c_ and the crystallinity of the lamellar stacks ω_c_^LS^ on the temperature, pressure and composition. As a result, the contribution *S*_a_^IL^(*T*,*P*) can be calculated with eqs 9 and 6, and then the total sorption *S* can be determined with eq 4.

In order to calculate the free energy of the interlamellar amorphous domains, we employ an approach which is similar to the one used by Flory^28^ to derive an expression for the free energy of polymer networks. For simplicity, the lamellar stacks are modeled as a sequence of alternating layers of crystalline polymer (the lamellae) and amorphous material characterized by a well-defined lamellar thickness *l*_c_ and interlamellar distance *l*_a_. Let *V* be the volume of a region of the amorphous domains included between two parallel crystalline lamellae. The relationship between *V* and *l*_a_ is simply *V* = *A*_Σ_*l*_a_, where *A*_Σ_ is the area of one lamellar surface facing the interlamellar domains. Although this quantity may change with temperature due to mass exchanged between the lateral lamellar surfaces and the free amorphous mass, in our model it must be a constant as the latter phenomenon does not take place (see discussion above).

**Figure 3:**
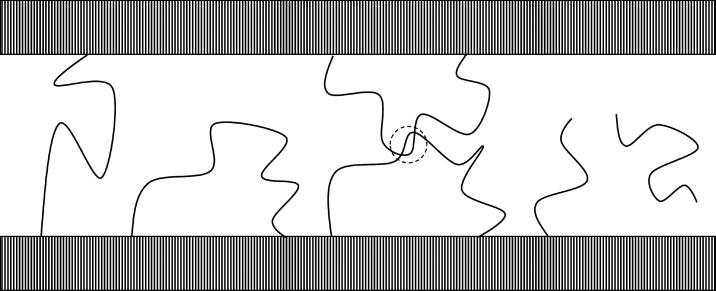
Schematic representation of the five types of polymer chains that can be found in the amorphous domains. From left to right: tie-chains, free (unentangled) loops, entangled loops, tails, and free chains.

The polymer chains found in the interlamellar amorphous domains can be divided into five categories based on topological arguments (see Figure 3). Tails and free chains can safely be neglected as their effect should be minimal except for very low-molecular-weight samples. Moreover, we introduce here the major approximation that the interlamellar domains are only composed of tie-chains. This is not realistic, as both entangled and untangled loops should be greater in number than tie-chains.^27,37,57,58^ However, at the level of approximation employed in the following development, accounting for the full compositional complexity of the interlamellar domains would only introduce unnecessary model parameters without adding physical insight. The interested reader can find the full treatment elsewhere;^59^ nonetheless, this approximation will be progressively justified at key points of the following discussion.

Quantities referring to tie-chains are hereafter denoted with the subscript “T”. Furthermore, tie-chains are assigned the same average properties, i.e., the same end-to-end vector and number of monomers. The introduction of chain length distributions in the theory is straightforward but not necessary to capture the main physical features of the problem,^59^ and it requires additional assumptions on the shape of the distribution. Lastly, the topology of the interlamellar domain—i.e., the number of tie-chains, loops etc.—is assumed to be preserved as long as the temperature remains sufficiently lower than the melting temperature, close to which recrystallization and structural reorganizations may occur.^37^

**Figure 4:**
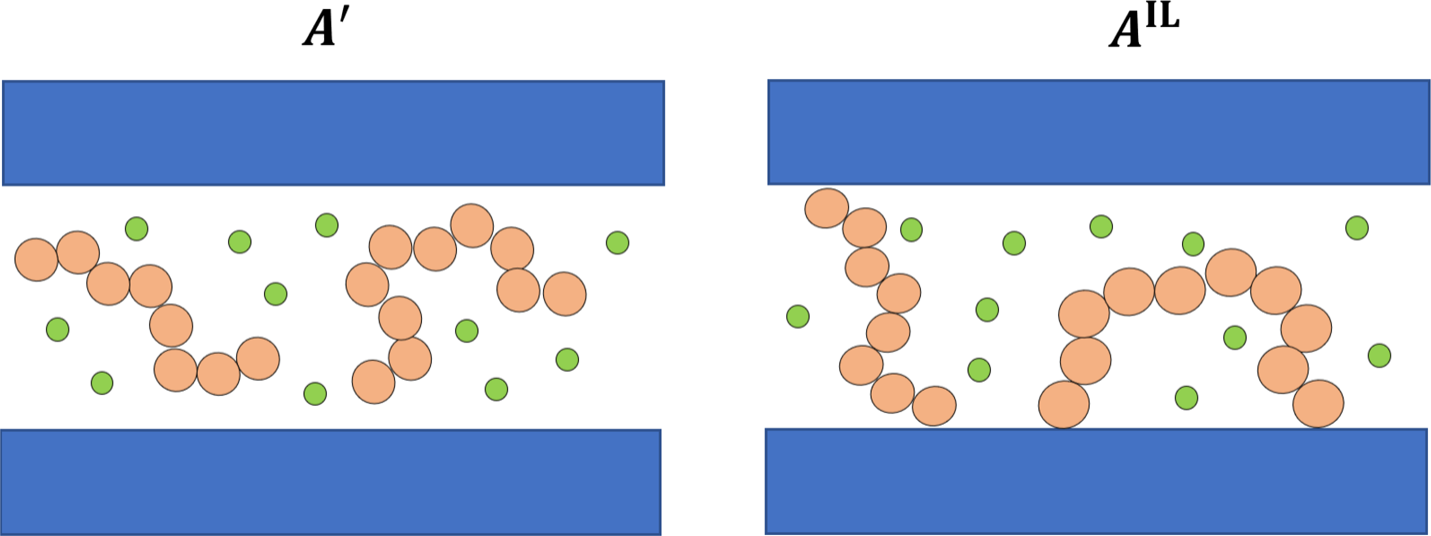
Schematic representation of the systems related by eq 24: the confined polymer–solute mixture (left panel) and the interlamellar domains (right panel). The orange chains represent polymer segments, the green circles solute molecules, and the blue rectangles lamellae. In our model, we assume that all the tie-chains have the same number of monomers and that loops are absent (in contrast to what is suggested in the picture).

By physically detaching all the tie-chains in the amorphous domains from the lamellar surface a confined polymer–solute mixture is obtained (the “confined fluid”—see Figure 4). The free energy of the interlamellar amorphous domains *A*^IL^ can be related to the free energy of the confined fluid *A*′ as follows:
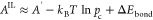
24Here, *ΔE*_bond_ is the energy gain due to the formation of irreversible chemical bonds between the tie-chains and the lamellae; *p*_c_, on the other hand, represents the probability of finding the detached chain segments in configurations compatible with bonding with the lamellae in the confined fluid; *k*_B_ is the Boltzmann constant.

The probability that a given tie-segment *j* in the confined fluid has the ends in positions **R**_*j*_^′^, **R**_*j*_^″^ can be written as 2*Δτ*^2^*p*_ee_^′^(**R**_*j*_^′^,**R**_*j*_^″^), where *p*_ee_^′^ is the end-to-end probability distribution in the confined fluid, and *Δτ* is the average volume within which the ends of the tie-chains remain confined due to thermal motion and bonding with the lamellae. This volume is in principle (at least) temperature dependent, but its variation with temperature is expected to be small due to the strength of the chemical bonds. Furthermore, one should note that in general *p*_ee_^′^ depends on temperature, solute concentration, and volume aside from the end-to end distance **R**_*j*_^′^ – **R**_*j*_^″^ and number of chain monomers *n*_T_. These dependencies are kept implicit for now to simplify the notation. The factor of 2 results from the equivalence of the two chain ends.

We then make the approximation
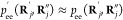
25where *p*_ee_ is the end-to-end probability distribution of a polymer chain with the same number of monomers in a *bulk* polymer mixture characterized by the same temperature, polymer, and solute density as the confined fluid.

With this approximation, one neglects the fact that the presence of the lamellae breaks the bulk symmetry, meaning that the actual probability for a free chain in the confined fluid of attaining configurations with its ends in a volume *Δτ* centered at **R**_*j*_^′^, **R**_*j*_^″^ might be different from 2*Δτ*^2^*p*_ee_(**R**_*j*_^′^, **R**_*j*_^″^) due for example to the interaction with the lamellae and the inability of tie-chain monomers to “cross” the lamellar surface. Nonetheless, if the tie-chains are generally taut—a hypothesis supported in the literature^37^—this approximation should be quite accurate.

Since *p*_ee_ is a bulk end-to-end distribution, due to translational and rotational invariance, we have
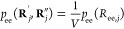
26where *R*_ee,*j*_ is the magnitude of the end-to-end vector of the *j*th chain. Since all of the the chains are assumed to have the same number of monomers *n*_T_ and (magnitude of the) end-to-end vector *R*_ee,T_, we can thus approximate *p*_c_ as
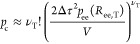
27where the factor ν_*T*_! accounts for the number of ways of choosing chain segments in the confined fluid to fill the ν_*T*_ pairs of bonding sites on the lamellae. This approximation amounts to assuming that as the structure of bound chain segments is progressively built from the detached chains, the probability for an unbound chain to attain configurations compatible with its target constrained state is not influenced by the presence of the other constrained segments. At this level, we are thus neglecting any pair and higher-order correlations between chains, as is customary in polymer networks models.^28,60^

Equation 24 for the Helmholtz free energy can therefore be rewritten as
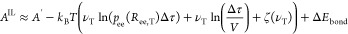
28Here, ζ(ν_T_) includes all the terms which depend only on the number of segments; since the topology of the interlamellar domains is assumed not to change, ζ(ν_T_) is just a constant in the free energy.

As a final approximation, we assume that the free energy *A*′ of the confined fluid is the free energy of a bulk polymer-solute mixture at the same temperature, volume, and composition:
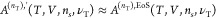
29Here, the superscript (*n*_T_) has been added to emphasize that the polymer chains in the confined and bulk mixture comprise *n*_T_ monomers (as in eq 19 and ensuing discussion). Approximating *A*′ with *A*^EoS^ amounts to neglecting finite-size effects on the thermodynamics of an unconstrained polymer mixture confined between two lamellae. This approximation is consistent with the assumption *p*_ee_^′^ ≈ *p*_ee_ discussed earlier. Equation 28 is thus now in the form of eq 10, and we can identify *ΔA*^c^ with
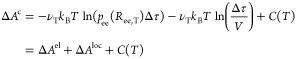
30where the quantity *C* (*T*) is volume-independent and contains the bonding energy Δ*E*_bond_ and the ζ(ν) term. *ΔA*^el^ is the “elastic” contribution to *ΔA*^c^ due to the stretching of the tie-chains. *ΔA*^loc^, on the other hand, represents an ideal gas term due to the loss of translational degrees of freedom of the constrained chains.

It can be shown^59^ that at the same level of approximation the inclusion of loops in the theory (both entangled or not) would lead to two additional stretching terms and two additional ideal gas terms to eq 30.

Before proceeding, it is useful to specify which state variables the free energy *A*^IL^ is characterized by. *A*^IL^ is a function of *T*, *V*, *n*_s_, and ν_T_ but also of the number of tie-chain monomers *n*_T_ and the (constant) constraints **Γ**_c_, which will be specified in the following section. Since the number of tie-chains ν_T_ is assumed to be constant, in the following we will thus write
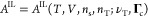
31to emphasize which variables are allowed to change and which are not. The same convention will be used for all of the other thermodynamic properties of the interlamellar domains.

#### 3.4.3 Constraint Pressure

Calculating the constraint pressure is now straightforward using eq 12:
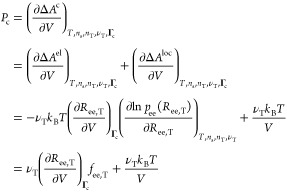
32Here, *f*_ee,T_ = −*k*_B_*T* (∂[ln *p*_ee_]/∂*R*_ee,T_)_*T*,*n*_s_,*n*_T_,ν_T__ is the (thermodynamic) force acting on the ends of the tie-chains under the assumptions of our model. The constancy of the constraints **Γ**_c_ in eq 32 influences how the partial derivative (∂*R*_ee,T_/∂*V*) is calculated. In the particular case of tie-chains, the constraints prevent the ends of the tie-chains from moving along the plane parallel to the lamellar surfaces. In our model, this condition is enforced by assuming that the projection of ***R***_ee,T_ on the lamellar surfaces is a constant, hereafter named δ_T_. Therefore, in the following, **Γ**_c_ ≡ δ_T_. Recalling *V* = *A*_Σ_*l*_a_ and using Pythagoras’ theorem for *R*_ee,T_, we thus obtain
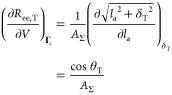
33where θ_T_ is the angle formed between ***R***_ee,T_ and the normal to the lamellar surfaces. This equation enforces one-dimensional swelling of the interlamellar domains, in line with other modeling studies.^32,36^ As mentioned in Section 3.2, many of the most common models developed for swelling in semicrystalline polymers^19,28,30^ assume isotropic swelling, which translates as
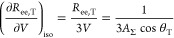
34Substitution of eq 33 in eq 32 yields
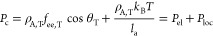
35where we have defined ρ_*A*,*T*_ = ν_*T*_ /*A*_Σ_ as the surface density of tie-chains on the lamellar surfaces. This form of *P*_c_ emphasizes how *P*_el_ is equivalent to the mechanical pressure that would be exerted on the interlamellar domains by substituting each tie-chain with a spring at tension *f*_ee,T_.

The presence of unentangled loops does not influence *P*_el_, as their end-to-end vector is not modified by variations of volume. Conversely, it can be shown^59^ that the presence of entangled loops gives rise to an additional term to *P*_el_:
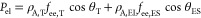
36Here, ρ_A,EL_ is the surface density of stems connected to entangled loops, whereas *f*_ee,ES_ and θ_ES_ are, respectively, the average force experienced by entangled segments—i.e., chain segments between two entanglements or between an entanglement and the lamellae—and their average angle with respect to the normal to the lamellar surfaces.

Due to the symmetry between the contribution of tie-chains and entangled loops to *P*_el_, it is apparent that neglecting entangled loops (as in our model) corresponds to representing a system of tie-chains and entangled loops as an equivalent system made only of tie-chains. The surface density ρ_A,T_ of tie-chains that appear in our model can thus be intended as the surface density of stems attached to an elastically effective molecule (tie-chain or entangled loop). The terms “tie-molecule” and “tie-segment” are now taken to refer to the individual tie-chains in the equivalent system, denoted by the subscript “T”.

Similarly, the mobility of the entanglement points and the localization in space of the loops give rise to additional “ideal gas” terms to *P*_loc_. In our model, these effects are neglected; however, a simple estimate suggests^59^ that *P*_loc_ should not be greater than a few MPa. Since the values of *P*_c_ reported indirectly^20,21^ are of the order of tens of MPa, we conclude that in our model *P*_el_ ≫ *P*_loc_ and the approximation *P*_loc_ ≈ ν_T_*k*_B_*T*/*V*—which neglects the contribution of loops—should thus not affect the description significantly.

In order to calculate *P*_c_, we need to specify *f*_ee,T_ and, therefore, the functional form of the bulk end-to-end probability distribution *p*_ee_(*R*_ee,T_). In general, this distribution is a complicated function of the end-to-end vector *R*_ee,T_ since it includes all of the effects of the intramolecular bonding interactions and of the interactions with the other monomers and the solute.

However, it is possible to show^29,31,33^ that for long chains in polymer melts or concentrated solutions, the average squared end-to-end distance of a chain with *n* bonds of length *l* is given by
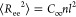
37where *C*_∞_ is the Flory characteristic ratio, which is roughly a constant for a given polymer.

In line with the phantom chain model used in most theories of polymer networks,^19,28,30,33^ we then assume that 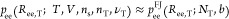
Here, *p*_ee_^FJ^ is the end-to-end probability distribution of an *equivalent* freely jointed chain with *N*_*T*_ monomers and a bond length of *b*. This approximation results in *p*_ee_ depending only on *n*_T_ and *R*_ee,T_, and therefore, *ΔA*^c^ is now composition-independent (in line with eq 15). The two equivalent chain parameters *N*_T_ and *b* can be found by enforcing that the contour length and mean square end-to-end distance of the two chains are the same:
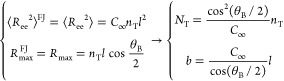
38where, *l* and θ_B_ are the bond length and bond angle of the real chain, respectively.

Using the accurate Langevin statistics—a rigorous result for freely jointed chains if the force is imposed instead of a displacement—we then approximate *p*_ee_^FJ^ with^31^
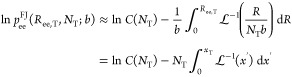
39where  is the inverse of the Langevin function , and *C* (*N*_T_) is a normalization constant (see Appendix A.3 for its definition). Here, we have defined the fractional extension of the tie-molecules *x*_T_ = *R*_ee,*T*_/(*N*_T_*b*). The force in the Langevin approximation is given by^33^
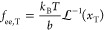
40Both eqs 39 and 40 reduce to the Gaussian approximation as *x*_T_ → 0. The key improvement offered by the Langevin approximation over the Gaussian treatment is that the former leads to a force that diverges as *x*_T_ → 1. This is important as tie-molecules should be fairly taut.^37^ Furthermore, it is shown later (cf. Section 4.2) that in order to comply with the local equilibrium hypothesis the Gaussian approximation leads to unphysical values of *x*_T_.

By substituting the expression for the force of eq 40 into eq 35, the constraint pressure then becomes
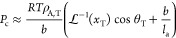
41As *x*_T_ → 0, the Gaussian approximation yields
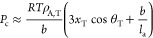
42In eqs 41 and 42, *k*_B_ has been substituted by *R*—the universal gas constant—by expressing ρ_A,T_ in mol m^–2^.

Before moving on to the next section, it is useful to summarize the assumptions and approximations leading to eq 41:

- In our model, the constraint pressure *P*_c_ emerges from an equilibrium statistical mechanical treatment, in which it is subtly implied that the interlamellar amorphous domains should be rubbery rather than glassy. In the latter case, the system could in fact be trapped in a local free-energy minimum. It may be possible to extend the theory to semicrystalline polymers with glassy amorphous domains using nonequilibrium theories such as the Non-Equilibrium Thermodynamics for Glassy Polymers (NET-GP) model originally developed by Doghieri, Sarti, and co-workers.^61,62^ The NET-GP theory has been successfully employed to describe sorption isotherms of semicrystalline polymers such as PTFE and MFA,^35^ with glass transitions occurring above room temperature.
- We assume that the interlamellar domains are only composed of tie-chains. Despite the crude approximation, we point out that the contribution to *P*_el_ is rigorously zero for unentangled loops and is symmetrical to the tie-chains’ contribution for entangled loops. After establishing that *P*_loc_ should be a minor contribution to *P*_c_, we then map the real system composed of tie-chains and loops to an equivalent one made only of tie-chains. This equivalent system is such that the surface density of the tie-chains ρ_A,T_ is equal to the surface density of elastically effective stems (i.e., belonging to tie-chains and entangled loops) in the real system.
- In order to find a simple expression for the free energy, the probability of finding a given chain segment in the confined fluid in configurations compatible with its bonded state is assumed to be independent of the presence of the other constrained chain segments. This assumption means that the molecular environment of the chain segments is not altered significantly upon the formation of the ties. This condition is violated if the chain segments in the interlamellar amorphous domains attain configurations that are atypical of the molten state.
- The effects that the surfaces of the lamellae have on the thermodynamics of the amorphous domains are neglected. In particular, the end-to-end probability distributions appropriate for concentrated polymer solutions are here used to approximate the probability of observing the two ends of a given tie-chain on the surfaces of two opposing lamellae. Furthermore, if the interlamellar distance *l*_a_ is small, finite size effects should make the free energy *A*′ of the polymer mixture confined in the interlamellar amorphous domains different from the one calculated using an equation of state for bulk fluids, *A*^EoS^.
- Langevin statistics is employed to approximate the end-to-end probability distributions, thus accounting for the finite extensibility of real chain segments. To our knowledge, this is the first time that such an approximation has been used instead of the Gaussian approximation in the context of predicting sorption in semicrystalline polymers. Furthermore, this assumption is consistent with the observation that tie-segments in the amorphous domains should be very taut.^5,37^

In order to calculate *P*_c_ and thus the equilibrium volume, it is necessary to specify the value of the unknowns of eq 41, namely ρ_A,T_, cos θ_T_, and *x*_T_.

#### 3.4.4 Equilibrium Volume: The Role of Morphology and Crystal Structure

The surface density ρ_A,T_ of stems connected to elastically effective chains can be conveniently expressed in terms of the surface density of stems ρ_A_ and the surface fraction *p*_T_ of stems that are connected to an elastically effective chain:
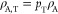
43The value of ρ_A_ can be inferred from knowledge of the crystal structure of the given semicrystalline polymer by “cleaving” the crystal along the (001) plane—i.e., perpendicular to the chain direction. It should be noted that in case the chain direction in the lamellae is not exactly normal to the lamellar surface—a phenomenon known as chain tilt^3^—the surface density of stems is decreased by a factor cos γ, with γ being the angle between the stem direction and the normal to the lamellar surface. In order to simplify the treatment, we set γ to zero so that cos γ = 1, as the angle should in general be small.^63^

**Figure 5:**
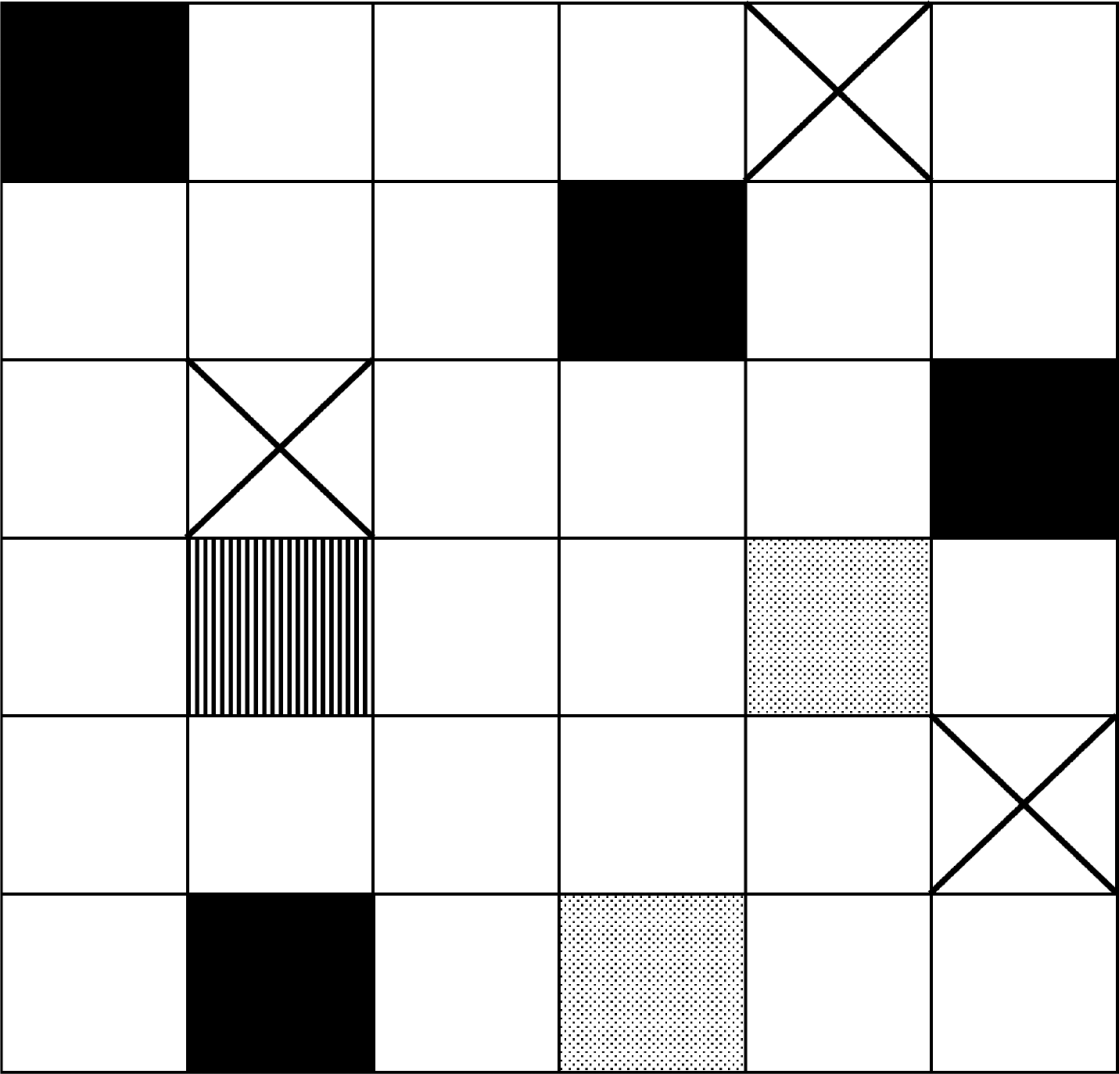
Schematic representation of the (001) crystal plane of a hypothetical polymer crystal with a square 2D lattice section. The direction of the chain segments in the lamellae is perpendicular to this plane. The empty white squares represent tight-fold sites on the lamellar surface; the filled black squares, stems connected to entangled loops; the crossed squares, tie-chains; the striped squares, tails; and the dotted squares, free loops. The surface fraction of elastically effective stems *p*_T_ is the ratio of the number of black and crossed sites to the total number of sites; in this illustration *p*_T_ = 7/36 ≈ 0.2. If *a* is the lattice parameter, for this square 2D lattice ρ_A_ = 1/*a*^2^.

On the other hand, *p*_T_ can be calculated as the ratio of the stems connected to a tie-chain or entangled loop on the lamellar surface and the total number of stems on that surface (Figure 5). The surface fraction of tie-molecules *p*_T_ is a morphological property of each particular semicrystalline polymer sample and can be affected by its thermal history;^37^*p*_T_ and similar quantities appear in many theories that aim to model the mechanical properties of semicrystalline polymers,^37,58,64−68^ and its value is usually obtained by correlating the experimental data with the model.

There is a large body of work dedicated to modeling the topology of interlamellar amorphous domains, with the aim of predicting the value of *p*_T_ or related quantities given the knowledge of, for example, the interlamellar spacing *l*_a_ and the molecular weight of the polymer. The most well-known examples of such theories are the Guttman and DiMarzio Gambler’s Ruin model^57,63^ and the Huang and Brown model.^58^ Despite their differences, all theories usually assume that the resulting chain topology after crystallization can be obtained by using the chain statistics in the melt, which is assumed to hold in the interlamellar domains.

This assumption has been questioned^37^ on the basis of chain reorganization at the mesoscale during crystallization or annealing. Due to the intrinsic complexity of semicrystalline polymer systems, it is unlikely that a predictive theory for sorption and swelling can be constructed using values obtained using such theories, unless more detailed models including the effects of cooling, chain defects, and molecular weight are proposed. In our present model, *p*_T_ is thus left as free parameter to be adjusted to best represent the experimental sorption data in order to gain *a posteriori* insight on the morphology of the sample.

The values of *p*_T_, cos θ_T_, and *x*_T_ are related. The equilibrium volume of the interlamellar amorphous domains is given by eq 14 as expressed in eq 44:
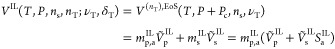
44Here, the extensivity of *V*_T_^(*n*),EoS^ has been used to express the volume in terms of the (state-dependent) partial specific volumes of the solute s and the polymer p in the interlamellar amorphous domains,  = (*T*,*P* + *P*_c_,*n*_s_, ν_T_). It is important to point out that the partial specific volumes are calculated using the reference polymer mixture with *n*_0_ monomers, which means that (cf. eq 20)
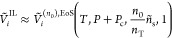
45with *ñ*_s_ = *n*_s_/ν_T_. The quantity
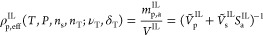
46can be identified as the effective polymer density in the interlamellar domains. We note that in the absence of penetrant molecules ρ_p,eff_^IL^ = ρ^(*n*_T_),EoS^(*T*, *P* + *P*_c_, 0, ν_T_) is simply the (mass) density of the pure interlamellar domains which in our model is slightly higher than the density of a pure polymer liquid—and consequently higher than the density of the free amorphous domains—due to the action of *P*_c_.

The interlamellar amorphous polymer mass *m*_p,a_^IL^ can in general be divided into two contributions: the mass of elastically ineffective molecules (tails and unentangled loops) *m*_p,NT_^IL^ and the mass of tie-molecules *m*_p,T_^IL^. *m*_p,T_^IL^ can be expressed as
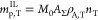
47where *M*_0_ is the molar mass of the monomer characterizing the polymer. Since *x*_T_ = *R*_ee,T_/*R*_max_ and 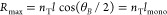
due to eq 38, the average number of tie-chain monomers *n*_T_ satisfies *n*_T_ = *R*_ee,T_/(*x*_T_*l*_mono_). By substituting for *n*_T_ and using *R*_ee,T_ = *l*_a_/ cos θ_T_, we can write
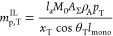
48If the polymer segments in the lamellae are chain-extended, ρ_c_^*^ = *M*_0_ρ_A_/*l*_mono_ is simply the mass density ρ_c_ of the lamellae, albeit temperature-independent. By combining this expression with eq 46, using *V* = *A*_Σ_*l*_a_ and *m*_p,a_^IL^ = *m*_p,NT_^IL^ + *m*_p,T_^IL^ the following expression is obtained:
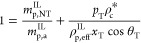
49After defining *f*_T_ = 1 – (*m*_p,NT_^IL^/*m*_p,a_^IL^) as the fraction of elastically effective polymer mass in the interlamellar domains (i.e., the free parameter in the Michaels and Hausslein theory, not to be confused with the average force *f*_ee,T_), eq 49 implies that
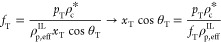
50or equivalently that
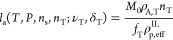
51

At fixed *T*, *P*, *n*_s_, and *n*_T_, the exact mass balance that we have just performed thus allows one to calculate the interlamellar distance (i.e., the volume) and the constraint pressure *P*_c_ simultaneously. At these conditions the interlamellar distance *l*_a_ is, in fact, only a function of *P*_c_ through ρ_p,eff_^IL^ (eq 46). On the other hand, since the lateral displacement of the ties δ_T_ is a constant, both *x*_T_ and cos θ_T_ are sole functions of *l*_a_ at fixed *n*_T_:
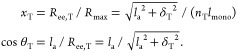
52It follows that at fixed *T*, *P*, *n*_s_, and *n*_T_, the constraint pressure given by eq 41 is only a function of *l*_a_. Substituting the expression for *P*_c_ in eq 51, an implicit equation for *l*_a_ is obtained, which can be solved to obtain the equilibrium interlamellar distance *l*_a_(*T*, *P*, *n*_s_, *n*_T_; ν_T_, δ_T_) and, thus, the equilibrium constraint pressure *P*_c_(*T*, *P*, *n*_s_, *n*_T_; ν_T_, δ_T_).

In our model, due to the absence of tails, free chains and unentangled loops we have *f*_T_ = 1. We have already pointed out that unentangled loops contribute negligibly to *P*_c_, and their effect only amounts to a scaling factor for the volume through *f*_T_ (cf. eq 51). The local equilibrium hypothesis provides additional support to this simplification. As mentioned in Section 3.3, this hypothesis postulates that each chain segment in the interlamellar domains is in equilibrium with respect to exchange of monomers with the crystalline lamellae. Equilibrium is established when the driving force of crystallization μ_p,mono_ is balanced by the variation of free energy arising from the increase of the number of monomers of each polymer segment (see eq 19 and Figure 2).

Since, at low temperatures, unentangled loops and tails have no way of resisting the driving force of crystallization, they should be almost fully incorporated in the crystalline lamellae—or at least accumulate in the boundary layer, becoming similar to tight-folds.^37,69^ This suggests *f*_T_ ≈ 1 at low temperatures. The condition *f*_T_ = 1 has been used implicitly by Mansfield^70^ and later by Albrecht and Strobl^52^ in order to build a model for premelting in PE; both studies assumed the local-equilibrium hypothesis and employed the Gaussian approximation for the end-to-end probability distribution of the chain segments.

Conversely, in models employing Michaels and Hausslein’s theory,^19,34,38−44^*f*_T_ is usually found to be much smaller than 1. Due to the aforementioned considerations, this finding appears to be inconsistent with the local equilibrium hypothesis at least for defect-free linear homopolymers. Nonetheless, it is possible that due to kinetic constraints or more sophisticated thermodynamic effects caused by defects, some loops or tails cannot crystallize, making *f*_T_ < 1.

#### 3.4.5 Chemical Potentials and Equilibrium

Now that the volume *V*^IL^ and free energy *A*^IL^ of the interlamellar domains can be calculated as functions of *T*, *P*, *n*_s_, and *n*_T_, it is sufficient to specify the dependence of the chemical potential of the solute μ_s_ and the monomer chemical potential of the polymer μ_p,mono_ on the external temperature *T* and pressure *P* in order to calculate *ñ*_s_ and *n*_T_ (see eqs 21 and 22). Knowledge of *ñ*_s_ and *n*_T_ immediately yields *S*_a_^IL^ as
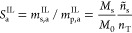
53where *M*_s_ and *M*_0_ are the molar mass of the solute and the polymer’s monomers, respectively.

The chemical potential of the solute imposed by the (pure) external fluid can be simply calculated with an equation of state:
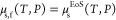
54On the other hand, by employing the definition of the specific Gibbs free energy of crystallization *Δg*_crys_, the monomer chemical potential imposed by the lamellae is given by
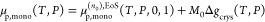
55Here, μ_p,mono_^(*n*_0_),EoS^(*T*, *P*, 0, 1) is the chemical potential *per monomer* of a pure polymer liquid (see eq 63 in Appendix A.1 for the definition) with *n*_0_ monomers per chain at the same conditions of temperature and pressure. *n*_0_ must be chosen to be a number high enough to make μ_p,mono_ a well-defined quantity due to the limiting properties of μ_p,mono_^(*n*_0_),EoS^ (see Figure 14 in Appendix A.1 and the related discussion). Below the melting temperature *T*_m_^0^ of a perfect polymer crystal *Δg*_crys_(*T*, *P*) can be approximated by
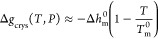
56where *Δh*_m_^0^ = *Δh*_m_(*T*_m_^0^) is the enthalpy of melting of a perfect polymer crystal at its melting point. In this approximation we neglect the difference in the heat capacity and specific volume between the pure crystalline polymer and the liquid polymer, as it is customary.^19,52,70^ More sophisticated models for *Δg*_crys_ can be obtained by considering the free energy of the lateral lamellar surfaces,^3,4^ which would make *Δg*_crys_ a function of the lamellar thickness *l*_c_.

Finally, the expressions for the two derivatives of  appearing in eqs 21 and 22 are (see Appendix A.2 and Appendix A.3 for the full derivation)
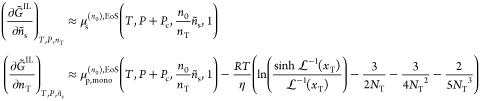
57Here, the constant η = *n*_T_/*N*_T_ is the ratio of the number of monomers and the equivalent Khun monomers of the chain *N*_T_ (cf. eq 38). As the superscript (*n*_0_) indicates, the chemical potentials are being calculated using the reference mixture (see eq 20 and the related discussion). The series in 1/*N*_T_ is the result of an asymptotic expansion of the normalization constant *C*(*N*_T_) in eq 39 and is arbitrarily truncated at third order.

In both the work of Michaels and Hausslein^19^ and in studies aimed at modeling premelting,^49−52^ the Gaussian approximation is used, which yields
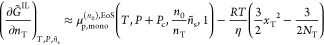
58It should, however, be noted that the increase in the monomer chemical potential due to *P*_c_ has not been considered in the aforementioned models for premelting.

The equality of the monomer chemical potential in eq 57 can be re-expressed using eq 55 as
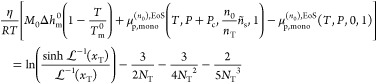
59This equation effectively determines the equilibrium fractional extension of the tie-chain monomers *x*_T_. One should note that the actual “size” *N*_T_ of the segments enters the equation only through the asymptoptic expansion in 1/*N*_T_ and through the dependence of *P*_c_ on *l*_a_ (and therefore *n*_T_, cf. eq 51). At temperatures sufficiently below the melting point of the polymer, however, the driving force of crystallization *M*_0_*Δh*_m_^0^(1 – *T*/*T*_m_^0^) is significantly bigger than both the asymptotic series and the difference in monomer chemical potentials on the left-hand side. By neglecting the asymptotic series on the right-hand side, it is therefore evident that the equilibrium fractional extension of the tie-chains *x*_T_ is determined only by the value of the driving force of crystallization at low enough temperatures.

This fact can be used to further justify the substitution of all entangled loops with tie-chains performed at the beginning of Section 3.2. It can be shown^59^ that including entangled loops explicitly in the description would have led to an equation similar to eq 59 for the fractional extension of the entangled segments *x*_ES_. The considerations just made thus imply that at a sufficiently low temperature we should have *x*_ES_ ≈ *x*_T_. Hence tie-chains and entangled segments have approximately the same fractional extension in the context of this model, possibly differing only in their average angle with respect to the normal to the lamellae. Since these angles are already averaged in the definition of the constraint pressure (see eqs 36 and 41), the substitution of all tie-entanglements with tie-chains assumed here should thus leave the qualitative features of the model unchanged.

The same arguments can also be invoked to show^59^ that as long as *T* ≪ *T*_m_^0^ or *l*_a_/*b* ≫ 1, the properties of the tie-chains (i.e., *x*_T_ and cos θ_T_) and therefore the constraint pressure *P*_c_ are very weakly independent of the interlamellar distance (see eqs 41, 50, and 59).

In conclusion, at each external temperature *T* and pressure *P*, our model allows for the equilibrium number of tie-monomers *n*_T_ and the equilibrium solubility *S*_a_^IL^ to be calculated through the combination of eqs 21, 22, 54, 55, and 57 or, equivalently, by minimizing the reduced potential Ω̃_s_^IL^ with respect to *ñ*_s_ and *n*_T_ (*cf*. Section 3.3 and eq 64).

### 3.5 Parameter Estimation

The prior knowledge and parameters needed to perform the calculations can be grouped in three main categories: an equation of state capable of representing the properties of both the external fluid and the polymer–solute mixtures, a number of polymer-specific parameters, and a few sample-specific parameters.

The equation of state is needed to calculate the chemical potentials of the solute and the polymer both in the amorphous domains (eqs 7 and 57) and the external fluid phase. Furthermore, the equation of state—and related parameters—must provide a reliable description of the volumetric properties (namely, partial specific volumes) of the target polymer–solute mixture in order to yield accurate estimates of the effective polymer density ρ_p,eff_ of eq 46.

The polymer-specific parameters (summarized in Table 2 for PE and isotactic PP), on the other hand, are needed in order to calculate both the properties of the equivalent Khun chains (i.e., *N*_T_ and *b*) and the specific Gibbs free energy of crystallization *Δg*_crys_ (eq 55). These parameters are readily available in the literature or can be calculated from literature data for the most common polymers.

**Table 2:** Polymer-Specific Parameters for Polyethylene and Polypropylene Used for All the Calculations^12,73−76^ (Monomer Molecular Weight Calculated per Main-Chain Bond)

property	symbol	PE	PP
bond angle	θ_B_	109.47°	109.47°
bond length	*l*	0.154 nm	0.154 nm
enthalpy of melting	*Δh*_m_^0^	293 J g^–1^	170 J g^–1^
melting temperature	*T*_m_^0^	414 K	460 K
surface stem density	ρ_A_	5.50 nm^–2^	2.86 nm^–2^
monomer molecular weight	*M*_0_	14 g mol^–1^	21 g mol^–1^
Flory characteristic ratio	*C*_∞_	6.9	5.9

Since in our current model *Δg*_crys_ only accounts for the difference in “bulk” free energy, *T*_m_^0^ should not be the melting point of the sample but rather the highest equilibrium crystallization temperature achievable for high-molecular weight, monodisperse, and defect-free samples at vanishingly small cooling rates. All real polymer samples will in fact melt at temperatures lower than *T*_m_^0^ due to the finite size of the lamellae and the presence of defects such as chain ends and branching.^4^ Similar considerations also apply to *Δh*_m_^0^.

Finally, a set of sample-specific parameters is needed to characterize the morphology of a given semicrystalline polymer sample. The model for the interlamellar domains first requires the specification of the fraction of stems on the lamellar surfaces attached to tie-molecules, *p*_T_, and their average lateral displacement δ_T_. While *p*_T_ is kept as a free parameters of the model, δ_T_ can be calculated if the interlamellar distance *l*_a_^*^ for a pure semicrystalline sample is known at a given temperature T* (and pressure P*). In fact, once T*, P*, *p*_T_, and *l*_a_^*^ are specified, δ_T_ is uniquely identified by combining eqs 51 and 59 and setting *n*_s_ = 0.

The specification of *p*_T_ and *l*_a_^*^ is therefore sufficient to fully characterize the interlamellar domains according to our model. In order to calculate sorption isotherms in a target sample, however, both the fraction of free amorphous mass ψ and the crystallinity of the lamellar stacks ω_c_^LS^ must also be specified (cf. eq 4). It is straightforward to show that
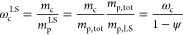
60Therefore, if ψ is kept as a constant free parameter, and the overall crystallinity ω_c_^*^ is known at temperature *T* *, it is possible to calculate ω_c_^LS,^*^^. However, the crystallinity of the lamellar stacks is not a constant due to mass exchanges between the lamellae and the interlamellar domains. There is, in fact, a relationship between ω_c_^LS^ and *n*_T_, obtained by using eq 47 and assuming *f*_T_ = 1:
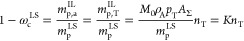
61Here, *K* is a constant due to the assumption that there is no mass exchange between the lamellar stacks and the free amorphous mass (i.e., that ψ is a constant). If T*, P*, *p*_T_, and *l*_a_^*^ are specified, it is possible to calculate *n*_T_^*^ and consequently *K* once ω_c_^*LS*,*^ is known. The crystallinity of the lamellar stacks can then be calculated at each condition of temperature, pressure, and composition via eq 61 by relating it to *n*_T_.

In conclusion, each semicrystalline polymer sample is uniquely characterized at an arbitrary temperature T* (for example, 25 °C) by the measurement of the crystallinity ω_c_^*^ and the interlamellar distance *l*_a_^*^, and by the two free parameters ψ and *p*_T_. As mentioned at the end of the previous section, however, at temperatures sufficiently lower than *T*_m_^0^ or when *l*_a_/*b* ≫ 1, the actual value of *l*_a_^*^ has very little influence on the properties of the interlamellar domains, apart for their actual linear dimension. Therefore, in most cases, if the interlamellar distance is not a property of interest, *l*_a_^*^ can be safely taken to be equal to a typical experimental value without appreciable changes in the predictions of the model.

The need for the inclusion of ψ in the model to better describe the experimental sorption data is made clearer in Section 4.3. We note that, in our work, ψ is kept constant, although just like ω_c_^LS^ this quantity may vary with temperature and during sorption.

## 4 Results and Discussion

### 4.1 Equation of State and Polymer-Specific Parameters

In order to calculate the chemical potentials of the solute appearing and the monomer chemical potentials of the polymer appearing in eqs 22, 21 and 57, the SAFT-γ Mie EoS^17,22^ is employed. In the SAFT-γ Mie approach, every molecule is modeled as a fully flexible heteronuclear chain. Each chain segment of type *j* represents a distinct functional group and interacts with other segments of type *k* through a four-parameter Mie potential; furthermore, each segment can be decorated with a number of different association sites that mediate a square-well interaction with compatible sites on other segments to represent short-ranged directional interactions such as hydrogen bonding.

SAFT-γ Mie is a group-contribution approach for molecules formed from different functional groups that are represented by the various segments. The like interaction parameters of each segment (*k* = *j*) are usually obtained by minimizing the deviation of the predictions of the equation of state from a set of target experimental data of molecules containing that segment. For example, the like interaction parameters of the water molecule modeled as a single segment can be estimated using vapor pressure and saturation-liquid density data of pure water.^71^ The unlike interaction parameters (*k* ≠ *j*) can be obtained in a similar manner using pure component (for molecules comprising different functional groups) or mixture data; however, some of the unlike group parameters can be obtained using combining rules^17^ in order to reduce the dimension of the parameter space.

In case the parameters for a given polymer or solute are not available, these could be obtained with the aforementioned parameter-estimation procedure using experimental data of the molten polymer, of the pure solute, and possibly of their mixtures.

The linear alkanes *n*-hexane and *n*-heptane are modeled as linear chains comprising two methyl CH_3_ groups and four and five methylene CH_2_ groups, respectively. Cyclohexane is modeled with six cyclic methylene cCH_2_ groups. The number of repeating units *n*_0_ of the polymer in the reference mixture needed to calculate the density and chemical potentials in eqs 57 and 49 is here fixed to *n*_0_ = 1000. Polyethylene is thus modeled as a linear chain comprising 1000 CH_2_ monomer groups, while polypropylene is a linear chain comprising 1000 CH_2_–CH(CH_3_) coarse-grained monomers.

The like SAFT-γ Mie interaction parameters between the CH_3_ and CH_2_ groups and the unlike dispersion interaction ϵ_CH_3_–CH_2__ were developed by Papaioannou et al.^17^ to reproduce pure component and mixture properties of linear alkanes. The like interaction parameters of the cCH_2_ group and its unlike interactions ϵ_CH_3_-cCH_2__ and ϵ_CH_2_–cCH_2__ with the CH_3_ and CH_2_ groups were determined by Dufal et al.^72^ to target the pure component properties of cyclohexane and some selected properties of mixtures of cyclohexane and other compounds. In our current work ϵ_CH_2_–cCH_2__ is modified slightly from Dufal’s 469.67 K to 471.85 K to better represent VLE data between cyclohexane and *n*-hexadecane.^86^ High molecular weight linear alkanes should in fact more closely match the properties of PE molecules.

Lastly, the like interaction parameters of the coarse-grained polypropylene monomer CH_2_–CH(CH_3_) were developed by Fayaz-Torshizi and Müller^18^ to reproduce the pure component properties of short, branched alkanes and the density of molten PP. The unlike energy interaction parameters between the PP monomer group and the CH_2_ and cCH_2_ groups are set to 339.34 K and 337.11 K, respectively; these values are determined by minimizing the difference between the SAFT predictions and experimental solubility data in aPP, as the latter is fully amorphous. All of the other unlike interaction parameters between the groups considered in our current work are obtained with combining rules (see the original SAFT-γ Mie papers^17,22^ for more details).

The polymer-specific parameters are obtained from the literature and are listed in Table 2. The specific enthalpy of melting of the perfect polymer crystal *Δh*_m_^0^ and the melting temperature *T*_m_^0^ of the semicrystalline polymer are approximations of the highest values reported for these quantities,^12,73−76^ since these values should more closely approximate the driving force of crystallization in the context of our model. The bond angles θ_B_ of both PE and PP are set to the tetrahedral value of 109.47° due to geometric considerations; although the true values of their bond angle deviate slightly from the tetrahedral angle, this difference does not impact the calculations significantly.

Similarly, for both polymers, the bond length is set to 0.154 nm—the typical length of a C–C bond between sp^3^ hybridized carbons. The monomer molecular weight *M*_0_ reported is calculated per bond: since the monomer of polypropylene is made of two distinct units (CH_2_ and CH(CH_3_)) with a total molecular weight of ∼42 g mol^–1^, *M*_0_^PP^ = 42/2 g mol^−1^ = 21 g mol^–1^. The surface stem density ρ_A_ is calculated using the lattice parameters of the orthorhombic unit cell of crystalline PE^77^ and of the monoclinic unit cell of the α form of crystalline isotactic PP.^78^

### 4.2 Pure Semicrystalline Polymer Properties

In this section, we assess the predictions of the model for a host of properties of pure semicrystalline polyethylene.

#### 4.2.1 Constraint Pressure and Fractional Extension

**Figure 6:**
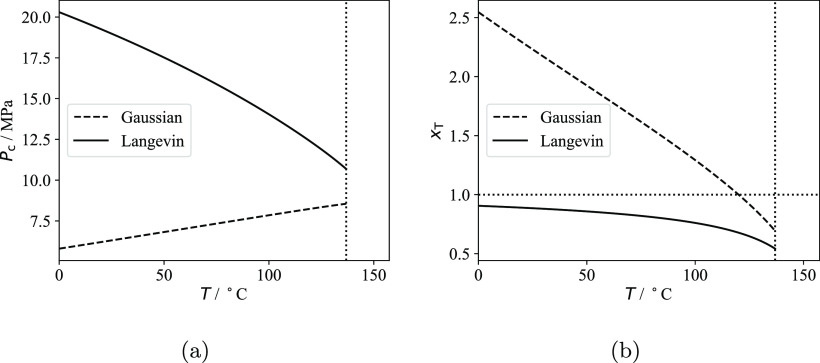
Temperature dependence of (a) the constraint pressure *P*_c_ and (b) the fractional extension *x*_T_ of the tie-segments in polyethylene using Langevin statistics and the Gaussian approximation. The vertical dotted line marks the (highest) melting temperature *T*_m_^0^ of polyethylene (see Table 2. Here, *l*_a_(25 °C) = 10 nm, and the surface fraction of elastically effective segments *p*_T_ = 0.3. The model parameters are summarized in Table 2. We note that the fractional extension calculated with the Gaussian approximation needs to exceed the maximum value of 1 in order to satisfy eq 58.

In Figure 6, calculations of temperature dependence of the constraint pressure *P*_c_ and the average fractional extension *x*_T_ of the tie-segments in the interlamellar amorphous domains of a hypothetical pure semicrystalline PE sample are shown using both Langevin statistics (eqs 41 and 59) and the Gaussian approximation (eqs 42 and 58). Here, the reference measurement temperature is T* = 25 °C and the corresponding interlamellar distance *l*_a_^*^ = *l*_a_(25 °C) is set to 10 nm due to the small influence it has on the intensive properties of the interlamellar domains. Furthermore, for illustration purposes *p*_T_ is set to 0.3, meaning that 30% of the stems on the lamellar surfaces are attached to a tie-chain or entangled loop.

As expected, the fractional extension of the tie-segments is seen to increase at low temperatures as a consequence of the dependence imposed by eqs 59 and 58. The use of the Gaussian approximation is found to yield unphysical values for the fractional extension *x*_T_ at low temperatures. Furthermore, while the constraint pressure increases at lower temperatures with the Langevin approximation, the contrary is true with the Gaussian approximation. In fact, *P*_c_ does not depend appreciably on *x*_T_ with the Gaussian approximation because the factor *x*_T_ cos θ_T_ appearing in eq 42 is approximately constant for *f*_T_ = 1 (see eq 50).

It is instructive to note that even if *f*_T_ ≠ 1 and was allowed to change in the Gaussian approximation, *P*_c_ would increase at lower temperatures only if *f*_T_ → 0 at low temperatures, as can be seen by substituting eq 50 into eq 42. This behavior is, however, impossible as it would imply that either the elastically effective segments are fully incorporated in the lamellae at low temperatures or that the elastically ineffective polymer mass (tails and loops) increases at lower temperatures, which is in stark contradiction with the local-equilibrium hypothesis.

As mentioned in the Introduction, Michaels and Hausslein’s theory^19^ implicitly predicts the presence of a constraint pressure (as noted by later authors^20,39^) and manages to predict the increase of *P*_c_ at low temperature despite using the Gaussian approximation. This is possible because in their work the swelling in the interlamellar amorphous domains is assumed to be isotropic, while here it has been postulated that swelling only occurs in the direction perpendicular to the lamellar surfaces (eq 33) due to the markedly one-dimensional nature of the lamellar stacks.

Since previous authors have found that *P*_c_ should increase at lower temperatures,^19,21^ we conclude that the use of the Langevin approximation (or any chain statistics that accounts for the finite extensibility of the chain segments) is necessary in order to develop a model relating the morphological properties of the interlamellar amorphous domains to their thermodynamic properties.

#### 4.2.2 Interlamellar Distance

**Figure 7:**
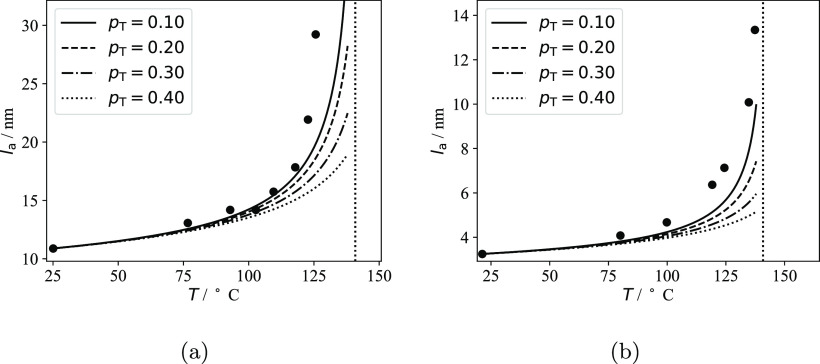
Temperature dependence of the interlamellar distance *l*_a_ in semicrystalline PE. The continuous curves represent calculations using the model developed with the Langevin approximation, and the symbols represent experimental data; the vertical dotted line denotes the melting temperature of the polymer *T*_m_ (see Table 2). The interlamellar distance at the lowest experimental temperature *l*_a_^*^ = *l*_a_(T*) is used to calculate δ_T_, while *p*_T_ has been adjusted to qualitatively reflect the experimental data: (a) data from Kavesh and Schultz;^79^ (b) Data from Tanabe et al.^53^ The predictions of the theory close to the melting point are not fully reliable due to irreversible changes occurring in the lamellar stacks.^79^

In Figure 7, the predictions of the variations of the interlamellar distance *l*_a_ in semicrystalline polyethylene obtained with our model are compared with experimental data taken from literature. In order to determine the theoretical curves, *l*_a_^*^ and T* are set to the experimental values at the lowest temperature while different values of *p*_T_ are considered.

It is apparent from Figure 7 that our model can be used to reproduce the experimental data semiquantitatively for temperatures which are sufficiently lower than the ideal melting temperature *T*_m_^0^ for any value of *p*_T_. The agreement is slightly worse for the data of Tanabe et al. shown in Figure 7b, possibly because the interlamellar distance is comparable to the Khun length of polyethylene, *b* ∼ 1.3 nm; approximating the real end-to-end probability distribution to that of a freely jointed chain in this limit may, in fact, lead to errors. At temperatures close to *T*_m_^0^, the smaller values of *p*_T_ appear to provide a better description of the experimental data, and it is tempting to conclude that *p*_T_ ∼ 0.10 for the samples assessed here.

However, it must be noted that at temperatures close to the melting point, irreversible transformations^19,80^ and structural reorganizations^37^ may change the topology of the lamellar stacks, violating the assumption inherent in our model that *p*_T_ should be a constant. Furthermore, the driving force of crystallization should change as the lamellae become thinner at higher temperatures: the lamellae are modeled as a continuum with a size-independent specific Gibbs free energy, whereas in reality lamellae of different thickness should have a different stability.^3,4^

Kavesh and Schultz^79^ have noted that for the sample considered in Figure 7a both the interlamellar distance and the lamellar thickness increased at temperatures higher than about 100 °C, indicating the presence of irreversible changes to the structure. In conclusion, the model is safely applicable only at low temperatures compared to *T*_m_^0^ where all values of *p*_T_ appear to provide a good agreement with the experimental data.

#### 4.2.3 Crystallinity

**Figure 8:**
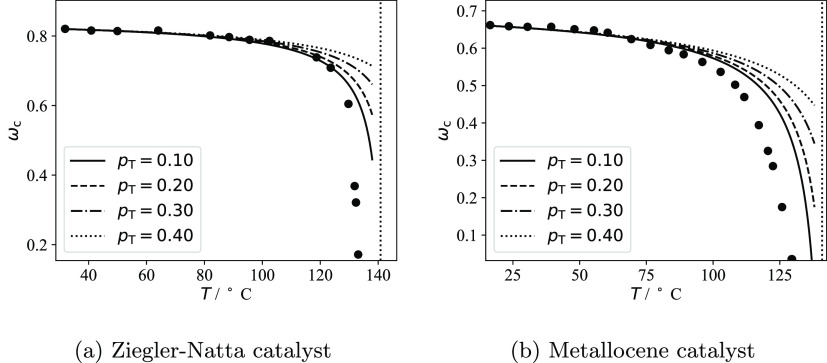
Temperature dependence of the crystallinity of different highly crystalline samples of PE. The continuous curves represent the calculations using our model developed with the Langevin approximation, and the symbols represent experimental data reported by Paricaud;^1^ the vertical dotted line marks the melting temperature of the polymer *T*_m_^0^ (see Table 2). The interlamellar distance at 25 °C is set to *l*_a_^*^ = 10 nm.

As discussed in Section 3.4.5, the model developed in our current work can be used to calculate the variation of the crystallinity ω_c_^LS^ of the lamellar stacks. For highly crystalline samples, all of the amorphous mass should be interlamellar,^81^ and the melting curves—i.e., plots of the crystallinity versus temperature—should thus be reproducible, provided that the temperature remains sufficiently lower than the melting temperature (as per the discussion in the previous section). The calculation with our model using various values for *p*_T_ are compared to the experimental melting curves of two highly crystalline polyethylene samples in Figure 8.

For all of the calculations, the crystallinity ω_c_^LS,^*^^ at the lowest experimental temperature T* is set equal to the experimental crystallinity at that temperature. For the same reasons highlighted earlier, the agreement between the model and the experimental data is satisfactory at low temperatures and deteriorates at higher temperatures. Similarly, the smallest values of *p*_T_ appear to provide better agreement at higher temperatures, where the predictions are expected to be less reliable.

In order to calculate the melting curves of polymer samples with low crystallinity, the temperature dependence of ψ must be known. The total crystallinity of the sample ω_c_ is related to this quantity via eq 60 once ω_c_^LS^(*T* ) is known. Here, ψ is assumed to be a constant, but in reality, it should change with temperature as pointed out in Section 3.4.1.

For instance, Flory^55^ and later Sanchez and Eby^56^ showed that the presence of noncrystallizable units in a polymer chain—e.g., branches, chain ends—causes the crystallinity of a polymer sample to be temperature dependent. Since a global thermodynamic equilibrium is assumed in these models, they are naturally better suited to describe the free amorphous mass which by definition is less constrained (i.e., more mobile) than the interlamellar polymer mass. It is therefore unlikely that our model can describe the melting curves of low-crystallinity samples. However, for the purpose of calculating the sorption isotherms, approximating ψ with a constant is reasonable as long as the temperature is sufficiently lower than the melting temperature. The changes in the crystallinity of semicrystalline polymer samples should in fact be small in this regime.^1^

### 4.3 Sorption of Simple Gases in Semicrystalline PE and PP

In this section, we optimize the values of the free parameters of our model, *p*_T_ and ψ, to reproduce the measured sorption isotherms of *n*-hexane, *n*-heptane, and cyclohexane in the six polyethylene and polypropylene samples considered. Since *p*_T_ and ψ are properties of a given individual polymer sample, a pair of values is assigned to each sample in order to minimize the difference between the experimental sorption isotherms and the calculations obtained with the model.

#### 4.3.1 Evidence for the Presence of Free Amorphous Polymer: ψ > 0

First of all, we provide evidence to support the necessity of including the free, unconstrained amorphous polymer in the description (i.e., ψ > 0) in order to reproduce the experimental sorption isotherms. If all of the amorphous mass is interlamellar, the total sorption *S* can be calculated from eq 4 by setting ψ = 0. Therefore, here the amorphous solubility *S*_a_ represents the solubility in the interlamellar amorphous domains *S*_a_^IL^, which—provided *p*_T_ and the interlamellar distance *l*_a_^*^ at a given temperature T* are specified—can be calculated with the help of eqs 6, 41, 51, and 57 at each temperature *T* and pressure *P* of the external fluid. Finally, the total crystallinity ω_c_ is simply the crystallinity of the lamellar stacks ω_c_^LS^, and the dependence on the temperature and amount of solute dissolved can be calculated with eq 61.

Apart from the two free parameters *p*_T_ and ψ, the crystallinity ω_c_^*^ at a given temperature T* and the polymer-specific parameters of Table 2, the value of *l*_a_^*^ = *l*_a_(T*)—the interlamellar distance at a given temperature T*—needs to be specified in order to calculate δ_T_ (the reader is referred to the discussion at the end of Section 3). While it is necessary to measure this quantity if its variations are to be calculated, the phase equilibrium and most other properties of the semicrystalline samples are almost independent of the precise value of *l*_a_ in the context of our model as discussed after eq 59. For all of our calculations of the phase equilibria, we set *l*_a_(25 °C) = 10 nm, which is a typical value for this quantity for both PE^79^ and PP.^4^

**Figure 9:**
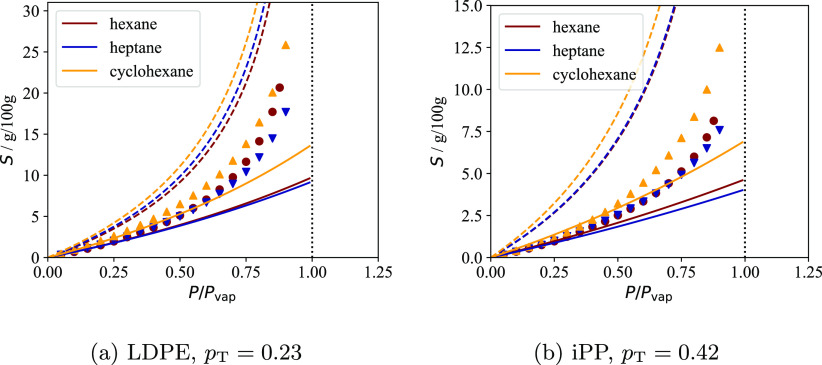
Sorption isotherms of *n*-hexane, *n*-heptane, and cyclohexane in the LDPE and iPP samples at 25 °C. The sorption (in grams of solute per 100 g of pure polymer) is plotted as a function of the total pressure *P* divided by the vapor pressure of the penetrant at that temperature. The continuous curves represent the predictions of the model, assuming that all the amorphous polymer lies between crystalline lamellae (ψ = 0); the value of *p*_T_ has been adjusted to capture the low-pressure behavior, while *l*_a_(25 °C) = 10 nm. The dashed curves represent theoretical calculations assuming that the solute cannot penetrate the crystallites (eq 3) but that there are no constraints, i.e., ψ = 1 – ω_c_ (eq 7). The symbols represent the experimental data determined in our current work: up triangles for cyclohexane, down triangles for *n*-heptane, and circles for *n*-hexane. Uncertainties in the data are smaller than the symbols. Not accounting for the free amorphous polymer results in an underprediction of the sorption when the pressure of the external gas approaches its vapor pressure. On the other hand, not accounting for constraints at all results in a systematic overprediction of the sorption.

In Figure 9 predictions of the model with ψ = 0 (continuous curves) are compared to the experimental sorption data of the three solutes (*n*-hexane, *n*-heptane, and cyclohexane) in the samples of LDPE and iPP. The value of *p*_T_ is adjusted for each sample to reproduce the low-pressure sorption behavior of the three penetrants simultaneously. The optimal values of *p*_T_ are 0.23 for LDPE and 0.42 for iPP. We present additional calculations (dashed curves) assuming that the crystallites are impermeable to the solute (as in our current model, eq 3) but also that all the amorphous mass is unconstrained (ψ = 1 – ω_c_); the equilibrium value of *S*_a_ = *S*_a_^F^ is thus the variable defined as *S*_a_^EoS^ in Section 3.1 (see eq 7).

As expected (see Figure 1), neglecting the presence of constraints results in a systematic overprediction of the experimental isotherms. On the other hand, if all the amorphous mass is subject to constraints—or, equivalently, if all the amorphous mass is interlamellar (continuous curves)—the curvature of the calculated sorption isotherms decreases significantly and the sorption at pressures close to the vapor pressure of the penetrant is systematically underestimated when the low-pressure behavior is captured correctly. While this behavior is found for every polymer sample, the curvature of the experimental amorphous solubility decreases with increasing crystallinity as highlighted in Figure 1.

This suggests that the higher the crystallinity, the more closely the morphology of the semicrystalline sample resembles the lamellar stacks model with ψ = 0; conversely, the lower the crystallinity, the closer the experimental sorption isotherms are to the model calculations with ψ = 1 – ω_c_ (dashed curves). This finding is consistent with the experimental observation that the free amorphous content should decrease with increasing crystallinity.^5^ It is thus to be expected that if ψ and *p*_T_ are simultaneously optimized to reproduce the experimental sorption isotherms, the optimal value of ψ should decrease with increasing crystallinity for all of the PE and PP samples.

#### 4.3.2 Sorption Isotherms Calculated with the Complete Model

**Figure 10:**
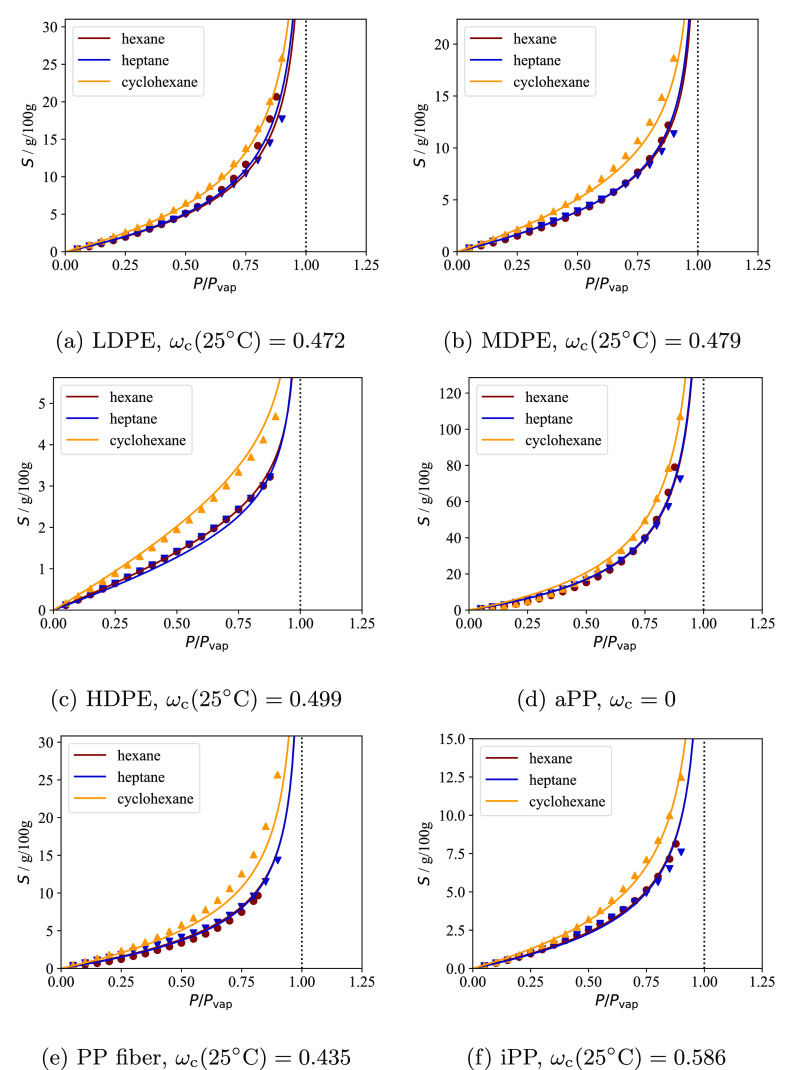
Sorption isotherms of *n*-hexane, *n*-heptane, and cyclohexane in the six polymer samples tested at 25 °C. The sorption (in grams of solute per 100 g of pure polymer) is plotted as a function of the total pressure *P* divided by the vapor pressure of the penetrant at that temperature. The continuous curves represent the calculations with the model after adjusting *p*_T_ and ψ for each polymer sample to best reproduce all three sorption isotherms simultaneously; the optimal values are reported in Table 3. The symbols represent the experimental data determined in our current work, with uncertainties which are smaller than the symbols.

The calculations obtained with the model after optimizing *p*_T_ and ψ simultaneously for each polymer sample (continuous curves) are shown together with the experimental sorption data (symbols) in Figure 10. The optimal parameters for each sample are listed in Table 3 together with the crystallinity at 25 °C calculated using the density measurements (eq 1). With the inclusion of the free amorphous polymer in the description (ψ ≠ 0), the agreement between the description of the model with the optimized parameters and the experimental data is excellent for all of the samples over the entire pressure range. Since atactic polypropylene is fully amorphous, we neglect any type of constraint in the amorphous mass (i.e., all amorphous mass is free) in the calculations by setting ψ = 1 and ω_c_^LS^ = 0 in eq 4.

**Table 3:** Sample-Specific Parameters^a^

polymer sample	ω_c_(25 °C)	*l*_a_(25 °C) (nm)	*p*_T_	ψ
LDPE	0.472	10	0.311	0.210
MDPE	0.479	10	0.288	0.106
HDPE	0.499	10	0.374	0.022
aPP	≈0	–	–	≈1
fPP	0.435	10	0.524	0.168
iPP	0.586	10	0.568	0.107

a The crystallinity at 25 °C is calculated with eq 1 and knowledge of the density of the samples. The inter-lamellar distance at 25 °C is set to 10 nm for all samples due to its negligible influence on the sorption isotherms. The values of *p*_T_ and ψ are obtained by minimizing the relative deviation of the experimental data of Figure 10 and the model calculations.

The good description of the experimental data for aPP obtained with the model confirms that the equation of state is well-suited to describe the PP–penetrant mixtures at 25 °C. As expected, for the semicrystalline samples the optimal value of ψ decreases with increasing crystallinity.^5^ Furthermore, it is apparent that the values of *p*_T_ for different samples of the same polymer are similar, with the average value of *p*_T_ being ∼0.32 for the PE samples and ∼0.55 for the (semicrystalline) PP samples.

The values of *p*_T_ obtained with the parameter-estimation procedure are higher than other estimates reported in the literature.^27,57,63^ In particular, Nilsson and co-workers^27^ have found that if the interlamellar chain topology is the result of random-walk chains between the lamellae, then the fraction of stems attached to elastically effective chains (i.e., tie-chains and entangled loops) should be ∼10% or *p*_T_ ∼ 0.1. Furthermore, Guttman^57^ and Flory and Yoon^69^ found that the fraction of stems performing tight folds *p*_TF_ should be ∼0.66 and ∼0.7, respectively. If *p*_NT_ is the fraction of stems connected to unentangled loops and tails and *p*_TF_ is the fraction of stems performing tight-folds, *p*_NT_ = 1 – *p*_TF_ – *p*_T_.

The combination of these consideration and our findings implies that for PE samples there should be very few stems connected to tails and unentangled loops since *p*_T_ ∼ 0.32. This is consistent with our current model, since due to the local equilibrium between the lamellae and the interlamellar amorphous chains *f*_T_ is assumed to be ∼1—which implies *p*_NT_ ∼ 0. However, the very high values of *p*_T_ obtained for the semicrystalline isotactic PP samples is inconsistent with the estimates *p*_TF_ ∼ 0.6—0.7.

Though this discrepancy could be due to one of the approximations employed to derive the model, the very high values obtained for *p*_T_ in the PP samples can be explained by the absence of free loops in the interlamellar amorphous domains. It is apparent by substituting cos θ_T_ from eq 50 in eq 41 that the same constraint pressure can be obtained by reducing the value of *p*_T_ and allowing *f*_T_ to be smaller than 1. In other words, if all of the interlamellar amorphous mass is either tie-chains or entangled loops at a fractional extension *x*_T_ (approximately fixed by eq 59), the average angle of the tie-segments with the normal to the lamellae must be closer to 90° than that with the presence of some elastically ineffective mass (see eq 50). As a consequence, if *f*_T_ is closer to 1, the tie-segments are more tilted with respect to the normal to the lamellae, and thus the component of the force normal to the lamellae (which contributes to *P*_c_) is smaller.

The high values of *p*_T_ obtained for PP may thus be an indication that tails and free loops must be present in the interlamellar domains of isotactic PP. This may indicate that isotactic PP is also a crystal-fixed polymer like sindiotactic PP:^4^ in the absence of the local-equilibrium hypothesis, more loops and tails can survive in the interlamellar domains, therefore making *f*_T_ < 1.

On the other hand, it could also be argued that the statistical arguments employed by Flory and by Yoon,^69^ Guttman,^57^ and Nilsson^27^ in order to estimate *p*_T_ and *p*_TF_ are not rigorously valid. All these estimates assume that, one way or another, the isotropy of the molten state is conserved after crystallization, generating the topology observed in the interlamellar domains. However, one could argue that only the amorphous chains segments that survive in the interlamellar amorphous domains are those that can resist getting incorporated in the lamellae (during or after crystallization) for topological reasons. It has been argued^82^ that, during crystallization, topological defects such as entanglements and tie-chains are segregated to the interlamellar domains due to their inability to crystallize, thereby increasing the value of *f*_T_ and *p*_T_ compared to the aforementioned estimates.

Owing to the nature of the approximations employed in our work, the values obtained for *p*_T_ and ψ are intended as qualitative estimates. The main purpose of our modeling approach is to determine sample-specific parameters that characterize the sorption behavior for each semicrystalline polymer sample regardless of the solute and temperature (as long as the latter is sufficiently lower than the melting point). The fact that a single pair of parameters can be assigned transferably to each sample to reproduce multiple sorption isotherms and that the optimal values of ψ and *p*_T_ conform to physically reasonable bounds—namely, 0 ≤ ψ ≤ 1 – ω_c_ and 0 ≤ *p*_T_ ≤ 1—is therefore encouraging and suggests that most of the essential physics of the system has been captured by the model despite the approximations performed.

#### 4.3.3 Effects of Temperature

**Figure 11:**
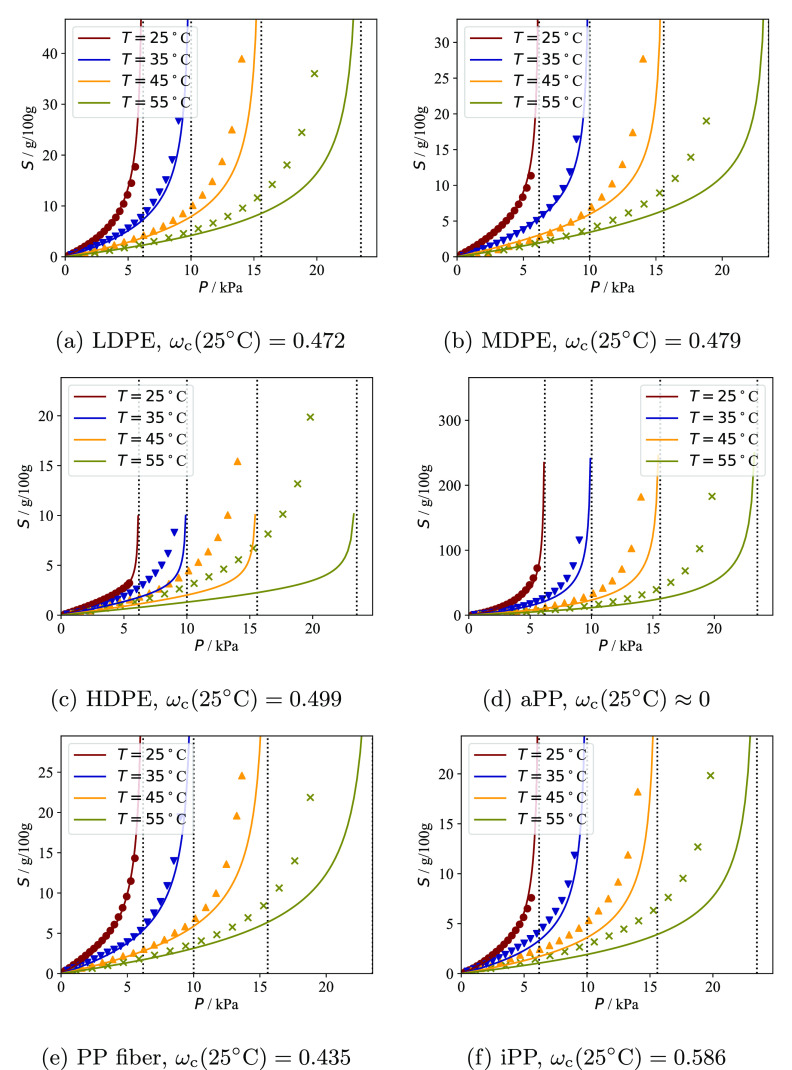
Sorption isotherms of *n*-heptane in six polymer samples at 25, 35, 45, and 55 °C. The sorption (in grams of solute per 100 g of pure polymer) is plotted as a function of the total pressure *P*. The vertical dotted lines denote the vapor pressure of *n*-heptane at the four different temperatures. The continuous curves represent the calculations with the model after adjusting *p*_T_ and ψ for each polymer sample to best reproduce the sorption isotherms of *n*-hexane,*n*-heptane, and cyclohexane at 25 °C (Figure 10 and Table 3). The symbols represent the experimental data determined in our current work, with uncertainties which are smaller than the symbols.

The sorption isotherms of *n*-heptane measured in the six polymer samples at 35, 45, and 55 °C are compared with the predictions of the model using the sample-specific parameter obtained for the isotherms of the three solutes at 25 °C. This comparison allows one to benchmark the validity of the assumption that ψ and *p*_T_ are temperature-independent, sample-specific properties. The calculations together with the new experimental sorption data determined in our current work are reported in Figure 11.

The solubility predicted with the model is seen to underestimate the experimental solubility at pressures close to the vapor pressure of *n*-heptane for the six polymer samples. This might be an indication that the variations of ψ with temperature must be accounted for in order to capture the temperature dependence of the sorption isotherms. It is important to note, however, that the fact that the solubility in the atactic PP sample—i.e., the one calculated neglecting crystallinity entirely—is markedly underpredicted at higher pressures and temperatures greater than 25 °C suggests that the systematic underprediction of solubility in all of the PP samples is due to the inability of the equation of state to describe the VLE properties of the mixture of *n*-heptane and PP at high pressures and temperatures. In other words, if the equation of state alone provided accurate predictions for the solubility of *n*-heptane in atactic PP at all temperatures, the quality of the predictions for the other two semicrystalline samples should improve.

Unfortunately, it is not possible to make the same argument for the PE samples as no fully amorphous PE exists at the temperatures investigated. Although sorption isotherms above the melting point of PE (*T*_m_ < 141 °C) could be used to gauge whether the equation of state properly describes the VLE properties of the mixture of *n*-heptane and PE, the comparison might be misleading due to the ∼80–100 °C temperature difference between these isotherms and the ones determined in our current work. Nonetheless, we believe that it is likely that the underprediction of solubility at high pressures in the PE samples is mostly due to the same systematic errors of the equation of state observed for the PP samples.

#### 4.3.4 Changes in the Interlamellar Domains during Sorption

**Figure 12:**
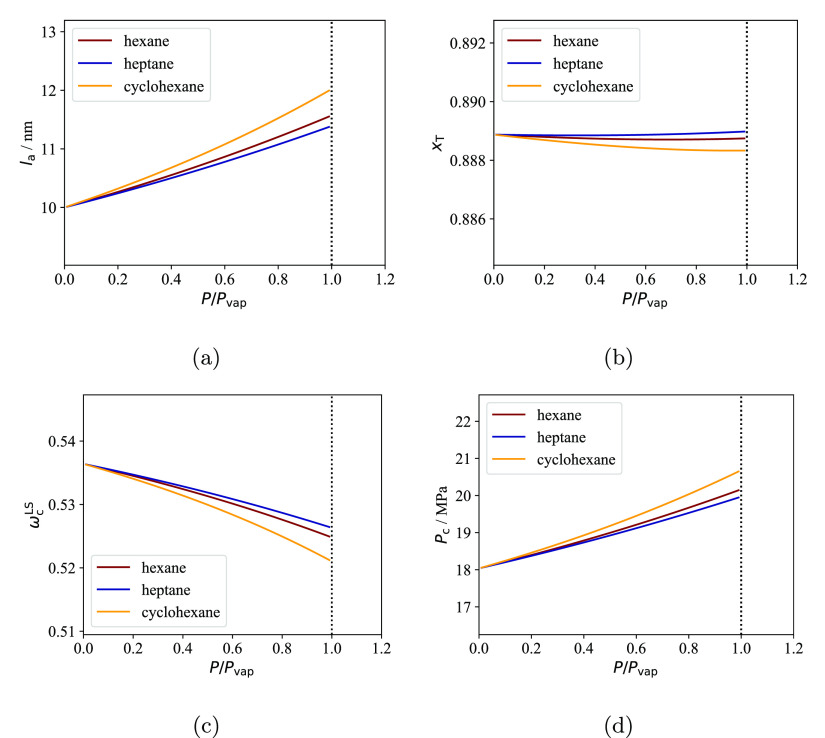
Variation of various properties of the interlamellar domains during the sorption process for MDPE at 25 °C, as calculated using the model developed with the parameters of Tables 2 and 3. All of quantities are plotted as a function of the ratio between the total pressure *P* and the vapor pressure of the penetrant at 25 °C as for Figure 10: (a) interlamellar distance *l*_a_; (b) fractional extension of the tie-segments *x*_T_; (c) crystallinity of the lamellar stacks; (d) constraint pressure. We note that the fractional extension *x*_T_ is almost a constant due to eq 59. The crystallinity of the lamellar stacks at zero sorption can be obtained by dividing the total crystallinity of the pure polymer by 1 – ψ: for MDPE, we have ω_c_^LS*^ = 0.479/(1 – 0.106) ∼ 0.536.

As mentioned at the end of Section 3.4.5, once the model parameters are specified, all of the properties of the interlamellar amorphous domains are state functions of the temperature *T*, external pressure *P*, and composition (in mass) *S*_a_^IL^. It is then possible to track the variations of these quantities along the sorption isotherms or with temperature at a fixed composition. As an example, the variations of *l*_a_, *P*_c_, ω_c_^LS^, and *x*_T_ along the sorption isotherms are shown in Figure 12.

The fractional extension *x*_T_ is found to be almost constant during sorption process as can be inferred from eq 59. At temperatures which are much lower than *T*_m_^0^, the lowering of the monomer chemical potential of the polymer μ_p,mono_^(*n*_0_),EoS^ due to the presence of the solute is insignificant compared to the driving force of crystallization, which is only a function of temperature. On the other hand, the swelling of the interlamellar domains (i.e., the increase of *l*_a_) causes the angle of the tie-segments with the lamellae to become closer to 0° and thus leads cos θ_T_ to increase. This explains the corresponding increase in *P*_c_ during sorption (eq 41). The variation of the crystallinity of the lamellar stacks is very limited.

#### 4.3.5 Swelling

The model developed allows one to quantify the swelling of the polymer sample. As shown in Appendix A.4, swelling can be calculated by means of
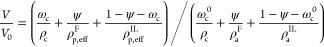
62Here, ω_c_^0^ and *V*_0_ are the crystallinity and the volume of the sample before sorption. Similarly, ρ_a_^F^ and ρ_a_^IL^ are the densities of pure free amorphous and interlamellar domains, respectively.

It should be noted that here we assume that the free amorphous mass does not change, i.e., ψ is constant. In general, the variation of ψ during sorption, if present, should be known in order to calculate the swelling of the sample. Nonetheless, the change in free amorphous mass due to sorption should be small (this is the case at least for the interlamellar domains; see Figure 12), and the assumption that ψ is a constant appears to be reasonable.

**Figure 13:**
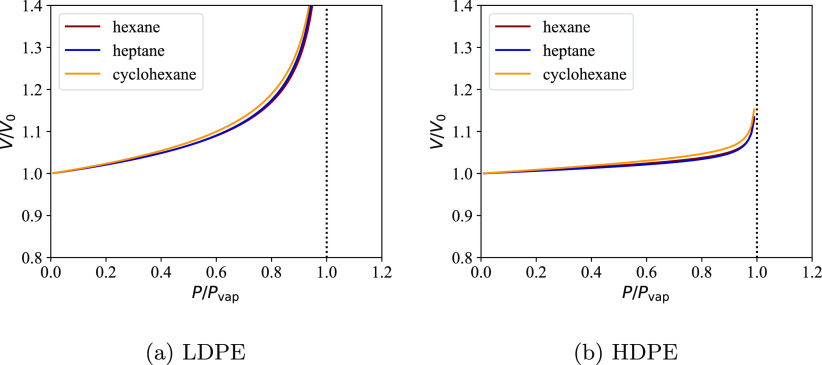
Swelling of the LDPE and HDPE samples during the sorption process at 25 °C as a function of the ratio between the pressure of the external gas and the vapor pressure of the penetrant at 25 °C. The continuous curves represent the calculations with the model obtained using eq 62. The model parameters for the two samples are listed in Tables 2 and 3. As expected, the LDPE sample swells considerably more than the HDPE sample due to the higher amount of penetrant dissolved in the free amorphous domains (see Figure 10).

**Figure 14:**
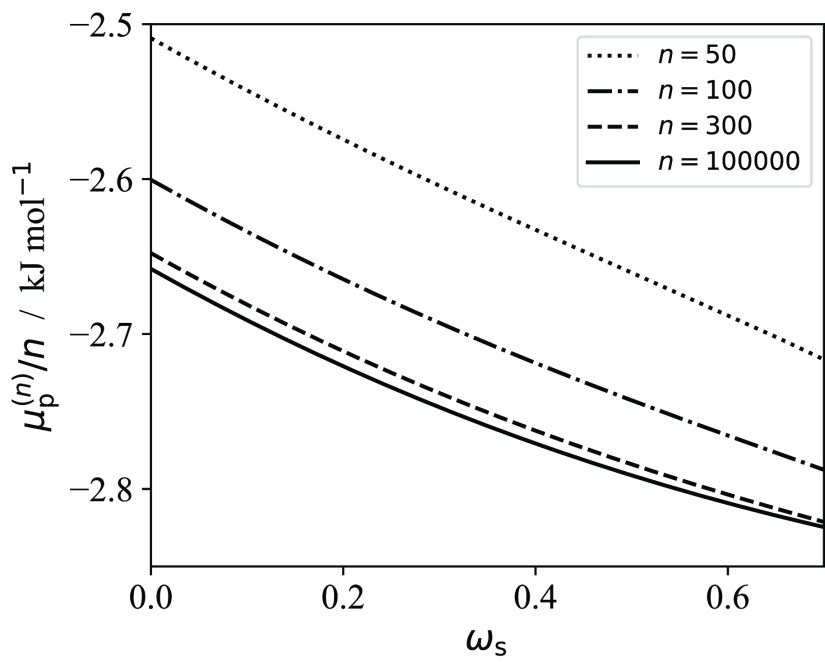
Dependence of μ_p_^(*n*)^/*n* in a mixture of *n*-heptane and polyethylene (see eq 63) on the mass fraction ω_s_ of *n*-heptane as a function of the number *n* of methylene monomers in the polymer, as calculated using the SAFT-γ Mie model of Papaioannou and co-workers^17^ at 300 K and 1 bar. We note that μ_*p*_^(*n*)^/*n* approaches the continuous curve (*n* = 100 000) very rapidly for *n* > 100.

In Figure 13, the swelling of the LDPE and HDPE samples during the sorption process of the three penetrants considered is determined at 25 °C. Unfortunately the swelling of the samples was not measured, preventing a direct comparison with the predictions. As expected, the LDPE sample swells significantly more then the HDPE sample because the former has a higher content of free amorphous mass and the solubility in the free amorphous domains is higher than in the interlamellar amorphous domains.

## 5 Conclusions

We have presented a thermodynamic model of semicrystalline polymers. Each polymer sample is assumed to comprise three distinct domains, in line with recent experimental observations:^5^ crystalline lamellae, interlamellar amorphous domains, and free amorphous domains. In our model, the free amorphous domains are treated as a subcooled polymer melt, and their mass fraction relative to the total polymer mass is denoted by ψ, one of the model parameters. The lamellar stacks, on the other hand, are assumed to be a sequence of alternating layers of crystalline lamellae and homogeneous interlamellar amorphous material with a well-defined boundary with the lamellae. A statistical-thermodynamic model of the interlamellar amorphous domains is developed in Section 3. The presence of tie-chains and entangled loops causes these domains to be (formally) subject to an additional constraint pressure *P*_c_ (eq 41). This additional pressure makes the solubility of any given penetrant lower in the interlamellar amorphous domains compared to the free amorphous domains, and it explains the experimental observation that the amorphous solubility in semicrystalline polymer samples is lower than the one determined by assuming that all the amorphous domains are subcooled polymer melts^19−21^ (see Figure 1).

The local-equilibrium hypothesis is implemented to explain the observed increase of *P*_c_ at low temperatures^21^ and the variation of the interlamellar distance with temperature.^53,79^ This allows one to determine the average number of monomers per tie-segment or equivalently its fractional extension as a function of temperature, pressure, and composition of the interlamellar amorphous domains.

To our knowledge, this is the first development of an explicit expression for the local-equilibrium hypothesis accounting for the finite extensibility of the chain segments via the Langevin approximation for the end-to-end probability distributions—previous authors invariably employed the Gaussian approximation.^19,49−52^ Furthermore, for the first time, we have unified in a single theory two seemingly unrelated phenomena—namely, the increase of *P*_c_ and lamellar thickness *l*_c_ with decreasing temperature.

The capability of the model in the calculation of a range of thermodynamic properties of the interlamellar amorphous domains and of the semicrystalline polymer in general is showcased in Section 4. In Section 4.2, we show that it is necessary to account for the finite extensibility of the chain segments (via the Langevin approximation) in order to explain the increase in *P*_c_ at low temperatures. The Gaussian approximation is found to give rise to unphysical values for the fractional extension *x*_T_ of the tie-segments. We are able to semiquantitatively predict the variations of the interlamellar distance and crystallinity of PE samples with high crystallinity at temperatures sufficiently lower than the melting temperature *T*_m_^0^ (Figures 7 and 8), regardless of the value of *p*_T_

The experimental sorption isotherms of *n*-hexane, *n*-heptane, and cyclohexane in three different PE samples and two PP samples are reproduced by adjusting *p*_T_ and ψ simultaneously for each sample. The experimental curves always lie somewhere between the theoretical curves for ψ = 0 and ψ = 1 – ω_c_ when the low-pressure behavior is captured (Figure 9). As expected,^5^ the optimal value of ψ decreases with increasing crystallinity for all samples tested. The optimal value for *p*_T_ is ∼0.32 for all the PE samples and ∼0.54 for the semicrystalline PP samples. The values obtained for PE samples are within the bounds obtained by previous authors^57,69^ who argued that the fraction of stems performing tight folds should be around 60–70%. On the other hand, the values of *p*_T_ obtained for semicrystalline PP samples are probably too high. We believe that this overestimation may be due to the assumption *f*_T_ ≈ 1, as the presence of nonentangled loops and tails in the interlamellar domains increases *P*_c_ at fixed *p*_T_ (see Section 4.3.2).

The assumption that *p*_T_ and ψ are temperature-independent sample-specific parameters is tested by comparison with experimental sorption isotherms of *n*-heptane in the six polymer samples at temperatures 25, 35, 45, and 55 °C (Section 4.3.3). For each sample, the two free parameters are optimized to reproduce only the 25 °C isotherms for *n*-hexane, *n*-heptane, and cyclohexane. At higher temperatures, the predicted solubility underestimates consistently the experimental solubility at pressures close to the saturation pressure of *n*-heptane, potentially due to the inadequacies of the equation of state.

Although here we have only compared the model predictions to sorption isotherms in which the external fluid is a gas (i.e., *P*/*P*_vap_ < 1), the same framework can be applied to studying the sorption isotherms of liquids or supercritical fluids in semicrystalline polymers. The only difference is that at equilibrium the sorption of liquids can be much higher than that of gases. A high amount of penetrant in the interlamellar amorphous domains may cause irreversible changes to the lamellar structure and thus invalidate the hypothesis that *p*_T_ should be a constant.

Similarly, the model can readily be extended to calculate the equilibrium sorption of multiple components at once in a given semicrystalline polymer. It would be interesting to predict how the interaction between different penetrants influences their equilibrium sorption at fixed temperature and partial pressure. This is certainly important in a practical setting as the fluids in contact with semicrystalline samples are very rarely pure. For example, typical reactor mixtures during the polymerization of polyethylene contain ethene, butene, nitrogen, and other hydrocarbons at the same time, and it has been shown that the presence of volatile gases in the reactor reduces the equilibrium sorption of ethylene in the polymerizing PE, thus slowing down the reaction.^83−85^

## A. Mathematical Derivations

### A.1 Monomer Chemical Potential

The polymer chemical potential *per monomer*—i.e., the “monomer chemical potential”—of a monodisperse polymer mixture is here defined as follows:

63

In this equation, *n* is the number of monomers comprising the polymer chains and μ_p_^(*n*),EoS^ is the “standard” chemical potential of the polymer in the mixture. μ_p,mono_^(*n*),EoS^ is independent of *n* when *n* → ∞, if the mass fraction of polymer is kept constant while taking the limit. As an example, in Figure 14, we show that, using the SAFT-γ Mie model of Papaioannou and coworkers^17^ for a mixture of *n*-heptane and polyethylene, the dependence of μ_p_^(*n*),EoS^/*n* on the weight fraction ω_s_ of *n*-heptane is not significantly influenced by the number of polymer repeat units for *n* > 100.

### A.2 Form and Derivatives of the Generalized Potential Ω̃_s_^IL^

The potential Ω̃_s_^IL^ = *G̃*^IL^ – μ_s_ ñ_s_ – μ_p,mono_*ñ*_T_ of the interlamellar domains is given by

64

Here, we have used the identity

65

the definition of constraint pressure (eq 32) and *V* = *A*_Σ_*l*_a_. *ΔA*^c^ and *P*_c_ are functions of pressure through the equilibrium interlamellar distance *l*_a_(*T*, *P*, *n*_s_, *n*_T_; ν_T_, δ_T_) (eq 51) and their respective definitions (eqs 30 and 41).

By substituting the unconstrained polymer mixture where polymer chains have *n*_T_ monomers with the equivalent mixture characterized by the same mass fraction of solute but in which polymer chains have a constant number of monomers *n*_0_ (see eq 20) and using the definition of monomer chemical potential (eq 63), we obtain

66

At the minimum of the potential Ω̃_s_^IL^ of eq 64, its derivatives with respect to *n*_T_ and *ñ*_s_ vanish:

67

This is equivalent to eqs 21 and 22. By realizing that  is a Legendre transform of  = *A*^IL^/ν_T_ with respect to *V*/ν_T_ and using eqs 30 and 65, we have

68

In order to obtain these results, the Gibbs–Duhem relations have been applied to cancel out some terms appearing when differentiating eq 65. The approximation in the first line of eq 68 becomes exact as *n*_T_ → ∞ for the reasons highlighted in Appendix A.1. By noticing that calculating the chemical potential at a volume *V* is the same as calculating it at pressure *P* + *P*_c_ (see discussion before and after eq 12) and using the reference mixture where polymer chains have *n*_0_ monomers, we have

69

### A.3 Derivatives of the End-to-End Probability Distribution in the Langevin Approximation

The only terms that needs to be calculated now is the partial derivative

70

appearing in eq 68. Here, the Langevin approximation (eq 39) has been employed, and in the following the subscript “T” of the tie-molecules will be dropped for clarity. Taking the derivative at constant volume translates immediately into a constant end-to-end distance condition due to eq 33 and the fact that the end-to-end distance is only a function of the interlamellar distance *l*_a_. We then need to evaluate the derivative

71

with *p*_ee_^FJ^ given by eq 39. Since *p*_ee_^FJ^ = *p*_ee_^FJ^(*R*_ee_, *N*; *b*), with *N* being the number of equivalent Khun monomers, we have

72

Here, we have defined the constant η = *n*/*N*, which can be evaluated using eq 38:

73

Let us thus concentrate on the derivative with respect to *N*. Using the properties of logarithms and eq 39, we have

74

We will first look at the second term on the right-hand side of this equation. By using the fundamental theorem of calculus and the properties of derivatives and remembering that the derivative is taken at constant *R*_ee_, we obtain

75

Let us define the fractional extension *x* = *R*_ee_/*Nb* and change the dummy integration variable to *x*′ to avoid confusion. Now, it is possible to show that if *f*(*y*) is a continuous invertible function on an interval [*a*, *b*], *f*^–1^(*y*) is its inverse, and *F*(*y*) its primitive, we have

76

where *c* is the integration constant. If we apply this lemma to eq 75 and note that the primitive *F*(*y*) of the Langevin function is

77

we obtain

78

Let us now evaluate the derivative d[ln *C*]/d*N* of eq 74. Since *C*(*N*) is the normalization constant of *p*_ee_^FJ^, by performing a change of variables we can rewrite *C*^–1^(*N*) as follows:

79

Differentiating, we obtain

80

Let us then define the function *g*(*x*) as

81

Its MacLaurin series is given by

82

The term of lowest order in the expansion, 3*x*^2^/2, is the only one accounted for in the Gaussian approximation. Now we use the properties of exponentials to perform the factorization

83

By using the Maclaurin series of the exponential function,

84

we obtain

85

Here, we have grouped the terms with the same power in *x*. In order to obtain the term of power *n*, the procedure is as follows:

- List all the possible ways in which one can obtain the number *n* by summing the degrees of the powers in *x* appearing in the MacLaurin expansion of *g*(*x*) – *c*_2_*x*^2^. For example, since the Maclaurin expansion of *g*(*x*) – *c*_2_*x*^2^ features only even powers greater or equal than 4, we can obtain the number *n* = 12 in four different ways: 12 = 4 + 4 + 4, 12 = 8 + 4, 12 = 6 + 6, 12 = 12.
- For each possible decomposition, write down a term obtained by multiplying factors *c*_*i*_ with multiplicity given by the decomposition. For example, write *c*_4_·*c*_4_·*c*_4_ = *c*_4_^3^ for 12 = 4 + 4 + 4 and *c*_4_·*c*_8_ for 12 = 8 + 4
- Multiply each term by (−*N*) to the power given by the total number of terms in the decomposition. For example, we multiply *c*_4_^3^ by (−*N*)^3^ and *c*_4_*c*_8_ by (−*N*)^2^
- Divide each term by a combinatorial factor obtained as follows: for each different *c*_*i*_ appearing in the term, multiply the factor by *k*_*i*_!, where *k*_*i*_ is the multiplicity of that *c*_*i*_ factor in the term. For example, we divide the term *c*_4_^3^*c*_6_^2^ appearing in the decomposition of *n* = 24 by (3!·2!) = 12
- Sum all the terms

Now, since *I*(*N*)is given by

86

using the expansion just obtained we have

87

All of the integrals appearing in the final line of the equation above can be related as follows. Using the Leibniz integral rule to differentiate under the integral and taking α = *c*_2_*N*, we have

88

The integral *Q*_0_(α) can be further split into the sum of two integrals:

89

The second integral in eq 89 is related to the error function and it can be shown that both the integral and its derivatives in α decay exponentially as α → + ∞. Furthermore, exploiting the parity of *e*^–*αx*^2^^ we get

90

Combining the equations, we then have

91

Here, we have defined , *a*_*k*_ = (2*k* – 1)!!/3^*k*^, and we have used the fact that *c*_2_ = ^3^/_2_. The two exclamation marks represent the double factorial (e.g., 5!! = 5·3·1).

We can now use this expression to evaluate the series in eq 87. Since some terms are multiplied by powers of *N*, a bit of care is required to group the terms according to their power in *N*. After performing this operation and stopping at third order in the expansion, we have

92

Here, we have used the little-o notation *o*(*N*^–3^) to indicate a term that is vanishingly small with respect to *N*^–3^ as *N* → + ∞. Notice that this quantity includes all the *B̃*_*k*_(*c*_2_*N*) terms generated by each integral in the series; all of these terms decay exponentially as *N* → + ∞, and will thus always be included in the little-o regardless of the order at which we decide to stop the expansion.

Substituting the values for *c*_*k*_, *a*_*k*_, writing *A*_0_(*c*_2_*N*) explicitly, and regrouping, we finally have

93

Since we have to calculate d[ln *I]*/d*N* = *I*′(*N*)/*I*(*N*), the same operation should be performed for *I*′(*N*) in order to find its asymptotic expansion in powers of *N*^–1^. Here, we note that we can obtain the expression for *I*′(*N*) by formally differentiating the asymptotic expansion of eq 93 with respect to *N*:

94

We finally obtain

95

Combining this equation with eqs 74, 78, and 80, we finally obtain eq 96:

96

Notice that the expansion in eq 95 does not converge to the actual value of d[ln *I*]/d*N* for any value of *N*, as we have placed all the terms that are exponentially decaying in *N* (i.e., the *B̃*_*k*_(*c*_2_*N*) terms) in the little-o symbol. The series must then be intended as an asymptotic series in *N*^–1^ for d[ln *I*]/d*N*:

97

Higher-order expansions are thus not guaranteed to reduce the error in the approximation for d[ln *I*]/d*N*, if *N* is small. The procedure can be generalized to any functional form for the end-to-end probability distribution, provided that the function multiplying *N* in eq 39 is only a function of the fractional extension *x* and is analytic in *x* around *x* = 0.

### A.4 Swelling

The total volume of a semicrystalline sample is the sum of the volumes of the crystalline lamellae and the free and interlamellar amorphous domains:

98

The volume of the interlamellar amorphous domains and of the free amorphous domains can be obtained using the properties of the partial specific volume:

99

At each temperature *T* and total pressure *P* of the external fluid, the partial specific volumes in the interlamellar amorphous domains are calculated using the equation of state at *T* and *P* + *P*_c_ for a polymer–solute mixture at composition *S*_a_^IL^(*T*, *P*). On the other hand, the partial specific volumes in the free amorphous domains are calculated at *T*, *P*, and *S*_a_^F^ = *S*_a_^EoS^(*T*, *P*) (eq 7).

Since the crystallites are assumed to be impermeable to the solute, the volume of the crystalline polymer can be expressed as *V*_c_ = *m*_p,c_/ρ_c_. By factoring out *m*_p,tot_ (the total polymer mass) in eq 98 and using eq 99, the following expression is obtained:

100

Here, the effective polymer density in the free amorphous domains ρ_p,eff_^F^ is given by

101

mirroring the definition of ρ_p,eff_^IL^ in eq 46. Since the total polymer mass *m*_p,tot_ does not change, by taking *V*_0_ and ω_c_^0^ as the volume and the crystallinity, respectively, of the pure semicrystalline polymer (*n*_s_ = 0), we obtain eq 62.

## Notes

Data underlying this article can be accessed on Zenodo at http://doi.org/10.5281/zenodo.7199129 and used under the Creative Commons Attribution license.
